# Biocompatible and Long-Term Monitoring Strategies of Wearable, Ingestible and Implantable Biosensors: Reform the Next Generation Healthcare

**DOI:** 10.3390/s23062991

**Published:** 2023-03-09

**Authors:** Tian Lu, Shourui Ji, Weiqiu Jin, Qisheng Yang, Qingquan Luo, Tian-Ling Ren

**Affiliations:** 1 School of Integrated Circuit and Beijing National Research Center for Information Science and Technology (BNRist), Tsinghua University, Beijing 100084, China; 2 Shanghai Lung Cancer Center, Shanghai Chest Hospital, School of Medicine, Shanghai Jiao Tong University, Shanghai 200030, China

**Keywords:** wearable biosensors, ingestible biosensors, implantable biosensors, biocompatibility, long-term monitoring, healthcare

## Abstract

Sensors enable the detection of physiological indicators and pathological markers to assist in the diagnosis, treatment, and long-term monitoring of diseases, in addition to playing an essential role in the observation and evaluation of physiological activities. The development of modern medical activities cannot be separated from the precise detection, reliable acquisition, and intelligent analysis of human body information. Therefore, sensors have become the core of new-generation health technologies along with the Internet of Things (IoTs) and artificial intelligence (AI). Previous research on the sensing of human information has conferred many superior properties on sensors, of which biocompatibility is one of the most important. Recently, biocompatible biosensors have developed rapidly to provide the possibility for the long-term and in-situ monitoring of physiological information. In this review, we summarize the ideal features and engineering realization strategies of three different types of biocompatible biosensors, including wearable, ingestible, and implantable sensors from the level of sensor designing and application. Additionally, the detection targets of the biosensors are further divided into vital life parameters (e.g., body temperature, heart rate, blood pressure, and respiratory rate), biochemical indicators, as well as physical and physiological parameters based on the clinical needs. In this review, starting from the emerging concept of next-generation diagnostics and healthcare technologies, we discuss how biocompatible sensors revolutionize the state-of-art healthcare system unprecedentedly, as well as the challenges and opportunities faced in the future development of biocompatible health sensors.

## 1. Introduction

Biosensors deployed on the skin, in the gastrointestinal tract (GI tract), and even in the body offer new opportunities to detect all types of body health information in the field. Basically, such sensors come in three forms: wearable (which typically function on the skin), ingestible (which are typically introduced into the gastrointestinal tract by swallowing or other actions), and implantable (which can function directly in the body, in most cases in direct contact with tissue).

These three types of sensors have different functions depending on where they are deployed, with the more developed being wearable biosensors, particularly skin-contact biosensors (or epithelial sensors), deployed on the skin surface to obtain important signs, such as heart rate (HR), respiratory rate (RR), blood oxygen saturation (SpO_2_), and blood pressure (BP), through pressure, photonics [1]. Ingestible biosensors, on the other hand, are usually in contact with the body’s mucosa (especially the GI tract mucosa), which makes them interact extensively with the body’s mucosal immune and GI tissues, posing potential risks, such as substance residues, biotoxicity, allergy, and chemical irritation. Implantable sensors, on the other hand, are usually in direct contact with human tissues, deployed in human brain tissue, blood system, adipose tissue, etc. This makes it possible to obtain some important physiological and biochemical parameters in situ, enabling the monitoring of some features related to internal lesions and infections in the early stages of the disease process, and the in situ continuous sensing of them, but their energy supply and miniaturization problems also limit their further development.

The performance limitations and potential risks of these biosensors when applied to the body have promoted advances in material biocompatibility, device reliability strategies, and their miniaturized size and need for long-term monitoring have also raised new issues in terms of energy supply, micro- and nano- integration on system design, material selection, and device processing. A number of recent studies have answered these questions from various aspects. In this review, we detail and summarize the successful answers to these questions by researchers worldwide. First, we present the needs of these sensors in terms of biocompatibility, mechanical compatibility, and energy supply in terms of the characteristics of the required functions of the three classes of biosensors, and summarize their sensing parameters and performance in turn. Second, long-term, reliable, and stable biosensors usually require a series of special design strategies, especially to address the power supply. Based on state-of-the-art technologies, we discuss the role of biocompatible sensors in driving the next generation of integrated diagnostic and therapeutic devices, as well as the transformation of healthcare delivery and smart management. Finally, the future and challenges of biocompatible sensors are also looked at in the concluding remarks.

## 2. Wearable Biosensors for Healthcare

Wearable sensors were originally developed to monitor a physiological indicator over time to provide information about human physiology outside of the hospital setting. In the past few years, wearable systems have shown high performance in monitoring body temperature, body movement, blood pressure, metabolites, and biomolecules. The connotation of wearable sensors is to detect signals from the human body in a lightweight, portable, reliable, and continuous manner, providing a variety of functions, including disease prognosis, changes in condition, and monitoring of vital signs. Although it has less complex contact with human tissues, the wearable sensor still works close to the body and is subject to challenges, such as human movement and complex external environments. Therefore, striking an artistic balance between biocompatibility, regular monitoring, light weight and portability, and accurate detection is always an essential issue in developing wearable biosensors.

### 2.1. Features of Wearable Biosensors and Designing Strategies

In this subsection, we concisely review the important compatibilities of wearable sensors for healthcare, including mechanical biocompatibility and immune biocompatibility, and after that, some other essential advanced design strategies will also be briefly introduced.

#### 2.1.1. Mechanical Biocompatibility

The wearable sensors require mechanical biocompatibility to form tight contact with the skin interface. Essentially, the concepts of mechanical biocompatibility are low modulus, light weight, high flexibility, and good stretchability, which ensures that signal acquisition can be achieved continuously and consistently without limiting active body movements (e.g., running, changes in prone position) and imperative mechanical movements (e.g., breathing). In general, mechanical biocompatibility is achieved with some of the following options: appropriate stretchable structures (such as springs, waves, and flexures), implementation of thin layers, and application of flexible materials (such as polyvinyl alcohol (PVA), polyethene terephthalate (PET), polyimide (PI), Polyethylene naphthalate (PEN), paper, and textile materials).

First, the devices are miniaturized and gain flexibility through carefully designed structural arrangements. The most common strategy involves “island-bridge” layouts, where conductive wires (bridges) interconnect high-performance but rigid functional components (islands) [2]. In contrast, several wire-layout design strategies have effectively converted rigid materials into tensile materials and maintained electrical properties. For example, serpentine structures consisting of periodic arcs and straight segments have been widely adopted for connecting rigid islands on top of soft elastomers. In addition, the use of pre-tensioned elastomeric substrates or lay-up techniques has also worked well. In recent years, the mechanical mismatch problem of wearable devices has been largely solved by introducing flexible materials such as organic polymers and gels. However, the mismatch problem at heterogeneous interfaces still exists, and the structural optimization of device layout remains a crucial strategy to improve mechanical biocompatibility [3].

Second, the implementation of thin layer designs to obtain the desired flexibility is obtained according to the Euler-Bernoulli beam theory. For example, single-crystal silicon nanofilms with thicknesses of 100–200 nm can be transferred from silicon-on-insulator (SOI) wafers to thin polymer substrates. This integration allows bending to small radii of curvature without fracture because the bending stiffness is reduced by several magnitudes [4]. Recent work has also reported methods for producing large-area organic or inorganic devices on ultrathin substrates, resulting in bending radii as small as a few tens of microns, even with materials with relatively large moduli of elasticity [5].

Third, elastic materials, especially low-dimensional materials represented by carbon nanotubes, graphene, MoS_2_, and black phosphorus, primarily enhance the flexibility of devices. Many commercial polymers and elastomers can be used as substrates for flexible and stretchable electronics. Silicone materials, an example of PDMS (Polydimethylsiloxane), as early flexible materials, have an elastic modulus similar to skin, thus enabling optimal skin-device contact, adhesion, and implantation (Martirosyan and Kalani, 2011). In addition to silicone, other polymeric materials, such as polyvinyl alcohol (PVA) films and polyethene terephthalate (PET), have been used as support matrices or protective layers to optimize skin-electrode contact. These materials have different thicknesses, elastic modulus, adhesive strength and other physical properties. However, the complexity of fabricating high-performance integrated circuits limits many of the advanced digital functions of epidermal devices, such as wireless communication, signal processing, and power transmission. With technology development, sensing devices have been gradually integrated into clothing [6], shoe insoles [7], sweat towels, and other everyday items. Some degradable and biocompatible biopolymers have also been used to prepare wearable medical systems. This brings people closer to the latest technology and highlights the integration of wearable medical systems with everyday life.

The combined application of these strategies can improve the mechanical properties of the devices at a reasonable level and prevent phenomena such as material delamination or local fracture similar to those of rigid electronic components. For example, the study by Gao et al. used EGaln, which remains liquid at room temperature and is an alloy of gallium and indium with high surface tension and high electrical conductivity, making it an ideal conductor for stretchable and flexible sensors. They designed a proper microfluidic channel to enhance the low gauge factor (GF) of the stretchable and flexible sensor using the EGaln conductor as a substrate and then applied photolithography to establish a relatively smooth microfluidic channel. The microfluidic channel using the injection of EGaln into the Ecoflex elastomer provided a sensor with high stretchability and conformality [8].

#### 2.1.2. Immune Biocompatibility and Other Desired Features

In real-time medical applications, there are conditions of direct contact or indirect contact between the wearable biosensors and the biological interface, so it is hoped that wearable medical equipment will not cause additional health threats and avoid restrictions on daily activities, which points out the significance of immune compatibility of wearable biosensors. Therefore, previously mentioned flexible materials have been shown to be immune compatible and have low toxicity, which does not cause inflammation on the skin surface. Compared with those biocompatible synthetic materials, natural biomaterials have more excellent biological characteristics (such as renewability, low cost, water-solubility, biodegradability, self-adherence, self-cleaning, etc.) [9] and provide an important platform for the production of a variety of hybrid materials with certain functions for their abundant active groups. This approach is more environmentally friendly and skin-friendly for long-term deployment and waste recycling. Some emerging studies have applied chitosan [10], natural pollen [11], etc., to physical or chemical parameter sensing applications. As biocompatible and biodegradable materials make long-term monitoring possible, all of these wearable devices have an upper limit application time due to the natural turnover cycle of epithelial cells (around two weeks).

Mechanical biocompatibility and immune biocompatibility are the basis of the safe and stable work of wearable healthcare systems. Based on realizing basic functions, some improved features have been gradually endowed to wearable biosensors. For example, some flexible patterning manufacturing methods can be used to achieve tattoo-like electronic skin, which can be easily prepared, deployed, and aesthetically unified. Tang et al. improved the electronic tattoo to make it more sticky and better able to hold shape, increasing the effectiveness of the electronic skin as an actual tattoo [12]. Likewise, a number of research groups have developed self-healing and transparent materials for flexible and stretchable electronics. Details can be found in these topical reviews [13]. These features together are pushing wearable healthcare systems towards practical applications.

### 2.2. Detectable Indicators of Physical Health

**Figure 1:**
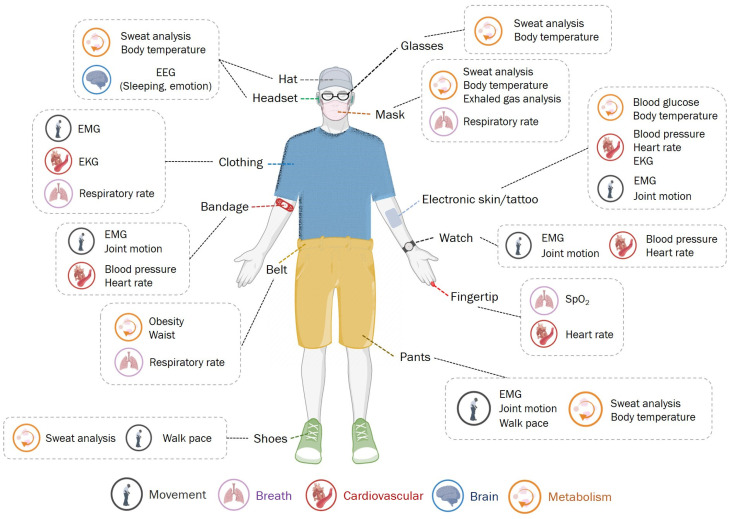
Scheme of detectable bio-signals on human body and their wearable detections (categorized by different part of human body and different systems that signals belong to) where the electrical activity signals that electrocardiography, electromyography, and electroencephalography are abbreviated as ECG (electrocardiogram) (or EKG), EMG (electromyography) and EEG (electroencephalogram).

As an initial idea, researchers tried to put old sensors on people’s bodies to get more accurate and compelling information. This leap has revolutionized the model of health testing. Some wearable electronic devices allow us to detect easily and in real-time (Figure 1). Understanding intrinsically related health parameters is essential when assessing an underlying disease’s health status and diagnosis. The research in the past decade has enriched the detection targets of the wearable medical systems, summarized in Table 1.

#### 2.2.1. Long-Term and Multi-Functional Monitoring of Vital Health Parameters

Heart rate, respiratory rate, blood oxygen saturation (SpO_2_), blood pressure, etc. are vital health parameters that directly reflect the human body’s basic physiological and pathophysiological status. Generally, abnormal fluctuations in vital health parameters are associated with trauma, infection, or some chronic diseases. Therefore, monitoring vital health parameters simultaneously is of great significance in both daily life and medical care.

An important vital health sign is the body temperature. Abnormal changes in body temperature range are effective indicators related to wound healing, cognitive status, cardiovascular diseases, and other symptoms. Therefore, it is necessary to detect temperature changes regularly in health management and clinical judgment. Flexible temperature sensors possess sufficient sensitivity and accuracy, which can be continuously measured by attaching different postures and motions directly to the non-planar skin surface under the smallest user perception. General medical devices, such as blood pressure monitors or stethoscopes, cannot realize multi-functional detection and are inconvenient to use due to their bulky and non-integrated design. Fortunately, the wearable healthcare system provides a multi-functional platform that is equipped with multifunctional sensors and has the advantages of portability, comfort, and aesthetics. A graphene-based strain sensor with the characteristics of high sensitivity, easy use, wearing comfort, a soft sensing patch, and integrated with a wireless Bluetooth unit was explored, which provided sensor interface to human skin and realized detection of both heart rate and blood pressure signals accurately making it a promising solution to home-based monitoring device [49]. Besides, the newest generation of the wearable device presented [50] can track bio-parameters including electrocardiogram, SpO_2_, skin temperature, and physical activity of the patient. In particular, it is worth mentioning that electronic skin/tattoos integrated with flexible electronic devices and systems became popular candidates for wearable personal medical applications. Based on various mechanisms, e.g., piezoresistive effect, piezo-capacitive effect, triboelectrification effect, thermal resistance effect etc., electronic skin with new materials and novel structures can detect almost all vital health signs with high performance. In Table 2, we listed materials, structures, performance (detect limit/response time/sensitivity), and mechanisms of several examples of electronic skin for detecting different targets of vital health signs. Especially, as the blood pressure can be estimated by the arterial pulse based on the pulse wave transmission time (PTT) [51], the electronic skin based on pressure sensing can realize to monitor the blood pressure and heart rate at the same time. Similarly, many other multi-functional wearable systems focus on monitoring vital health parameters and communicating with data platforms such as mobile phones for real-time, long-term data acquisition.

In addition, the detection of several other physiological parameters has been investigated. The introduction of smart contact lenses introduced biosensors into the eye, enabling physicians to see and monitor intraocular pressure, assisting in the diagnosis and treatment of various eye diseases, such as glaucoma [52]. In addition to IOP (intraocular pressure), various optical sensors employ a wide range of epidermal light attenuation techniques to measure everything from heart rate and blood oxygen (as seen in widely used consumer smart bracelets) to brain oxygen saturation and tissue health (e.g., detection of breast cancer) [53]. However, the optical noise and motion artefacts introduced by the environment must be controlled to achieve accurate measurements, the latter remaining a major challenge for wearable optical biosensors.

**Table 2:** Summary of tattoo for vital health signs detection.

Detect Target	Material	Structure	Performance	Mechanism	Reference
Blood pressure, Heart Rate	Ultrathin gold nanowires, thin polydimethylsiloxane (PDMS)	Sandwich	13 Pa/17 ms/1.14 kPa^−1^	Piezoresistive effect	[54]
Blood pressure, Heart Rate	Silver-flake, Eco-flex 00-30 silicone rubbers	Triangular-microprism	63 Pa/0.29 kPa	Triboelectrification effect	[55]
Blood pressure, Heart Rate	Graphene, PDMS	Hollow	1.2 ms/15.9 kPa	Piezoresistive effect	[56]
Blood pressure, Heart Rate	PDMS, Poly(3,4-ethylenedioxythiophene)–poly (styrene sulfonate) (PEDOT:PSS), Aqueous polyurethane dispersion (PUD)	Micro-pyramid array	23 Pa	Piezoresistive effect	[57]
Blood pressure, Heart Rate	PDMS, Polyethylene terephthalate (PET)	Micro-pyramid array	3 Pa/0.55 kPa	Piezo-capacitive effect	[58]
Blood pressure, Heart Rate	Silicon nanowire (SiNW)	Sandwich	3 ms/8.2 kPa	Piezo-capacitive effect	[59]
Respiratory rate, Blood pressure, Heart Rate	Graphene, PDMS	Random distributed spinosum	25.1 kPa	Piezo-capacitive effect	[60]
Body temperature	Silk-nanofiber-derived carbon fiber membranes (SilkCFM), PET	Graphitic local structure	0.81% per centigrade	Thermal resistance effect	[61]
Bode temperature	Thin and narrow gold	Filamentary serpentine mesh	Millikelvin precision	Thermal resistance effect	[62]

#### 2.2.2. Physiological Parameters

Body movement testing is important in rehabilitation medicine, inpatient observation, monitoring of people with disabilities, and assessment of movement levels. Periodic analysis of body movements can detect abnormal gait (e.g., freezing gait and forward gait in Parkinson’s disease; unsteady gait in Alzheimer’s disease, etc.) and sudden tremors of the hands (idiopathic or other pathological tremors), which are precursors to important degenerative diseases or manifestations of neuropathy due to chronic conditions, including Parkinson’s disease, Alzheimer’s disease, and diabetes mellitus, and contribute to the early diagnosis and treatment of these diseases.

On a mechanical level, body movements are usually manifested as larger strain changes on the skin and smaller strains identified by facial expressions, pulses, breathing, etc. The corresponding wearable systems can be appropriately designed according to the modalities and signal strength of different deployment sites. The connotation of human motion can also be extended to the vocal system, breathing, heart movement, and gastrointestinal digestive activity. There are already reports of intelligent artificial pharynxes [28] that can acquire subtle sounds in the pharynx, showing the potential for voice recognition and interaction. This could benefit laryngectomized patients and others with dysarthria, who could have lower learning costs and a more comfortable experience compared to patients with implanted vocal cord prostheses and esophageal speech. Such vibrating sensors could also be applied to the detection of respiratory, cardiac, and gastrointestinal sounds [63], allowing the study of implicit movements or movements within the human body.

In recent years, wearable skin sensors have provided a powerful platform for electrophysiological signal monitoring. Electrophysiological signals in neural and muscle tissue, such as electrocardiogram (ECG), electromyogram (EMG), and electroencephalogram (EEG), provide another dimension for measuring neurological disorders, cardiovascular disorders, and apparent motion. For example, EEG is a powerful tool for the development of new human-machine interface (HMI) and for the diagnosis of diseases related to brain function and neurological conditions, such as brain diseases, tumors, and sleep disorders. In electrical measurements, skin electrodes are used to extract depolarization signals from the heart muscle, which is known as an electrocardiogram. ECG provides general information about the cardiovascular system. Through the peak intensity, shape and period of ECG graphs, the skin-connected ECG sensor allows users to easily identify their heart condition and enables the early diagnosis of serious heart problems, such as cardiomyopathy, arrhythmia, and hypertension. Furthermore, when ECG is combined with other modalities, the hybrid sensing system may be able to present more information about human physical activities. As an illustration, Somayeh Imani et al. present a skin-worn wearable hybrid sensing system that offers simultaneous real-time monitoring of lactate and ECG signals, for more comprehensive fitness monitoring than from physical or electrophysiological detectors alone [11]. HMI with EMG as the control signal and skin current stimulation as feedback represents another important medical application in the field of robotics, artificial limbs, and machine-assisted living.

#### 2.2.3. Non-Invasive Detection of Biochemical Substances

Metabolites, such as polyols, uric acid, cholesterol, lactate, and glucose, can directly reflect the physiological activity of cells [64]. Their abnormalities can adversely affect the acid-base balance of the body, organ energy supply, and functional activity of organs. These substances are usually found in blood, tissue fluids, lymphatic fluids, and other body fluids. For example, sweat is rich in metabolite information and is often used as an ideal skin detection target for temporary tattoos and flexible skin patches [65]. Whether it is an optical sensor to detect sweat rate and pH, an impedance-based sensor to detect rate and conductivity, an ion-selective electrode to detect electrolytes, an amperometric enzyme sensor to detect metabolites, or a peel-based sensor to detect heavy metal analysis, wearable sweat biosensors can monitor various biochemicals and contribute to various physiological and clinical studies to monitor the health of patients/athletes [66]. A stretchable optical sweat sensor based on a thin and soft closed microfluidic system has been developed [67], which can collect sweat directly and rapidly without sweat evaporation or contamination, thus solving the traditional sweat challenge and allowing complex sweat sampling and measurement. In contrast, although saliva, urine, and tears also contain rich information on metabolites, wearable systems are often used as an alternative to the non-invasive detection of biochemicals as the convenience of sample collection is not well represented and poses a greater challenge to device design. However, recent advances in microelectronics, communications, and flexible substrates have enabled physicians to use smart contact lenses to obtain a variety of biochemical indicators of the eye. By analyzing the chemical composition of tears, smart contact lenses can provide real-time monitoring of glucose and lactate concentrations, helping to treat and prevent eye diseases [52].

Long-term detection is a major challenge to overcome for immune sensors. In 2017, the stability of antibody receptors was improved up to 96 h using human sweat as a platform for in vitro evaluation with room temperature ionic liquids to compensate for changes in sweat pH [68].

In addition, the synthesis of markers of some diseases can be detected by wearable devices. The most typical examples are cancer markers, such as prostate-specific function antigen (PSA, associated with prostate cancer) [69], human epidermal growth factor receptor (related to breast cancer) [70], etc.

## 3. Ingestible Biosensors for Healthcare

**Figure 2:**
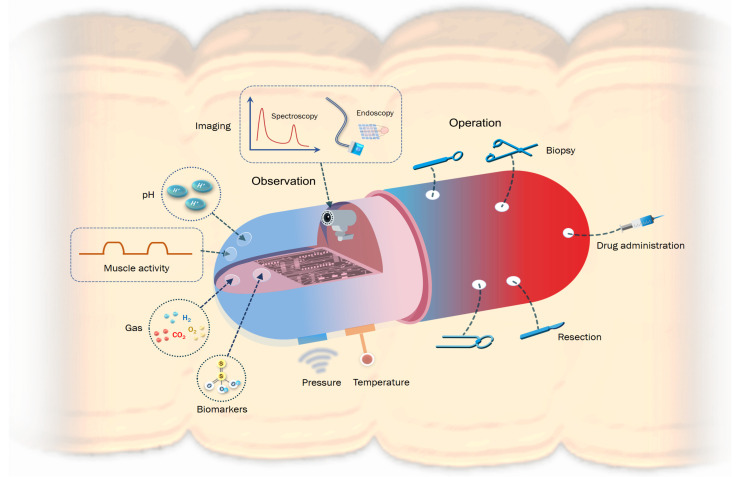
Schematic representation of ingestible sensor model working as a sensing and operational device for healthcare.

Wearable sensors cannot reach some niduses inside the body and detectable health indicators on the body surface are easily interfered with. As an alternative strategy, ingestible biosensors (Figure 2) which are called ingestible biosensing capsules (IBCs) can travel close to major organs through the gastrointestinal (GI) tract, monitor a vast range of biomarkers, serve as effective clinical tools for diagnostics, and even provide targeted surgical and pharmaceutical therapy. Ingestible sensors have long blown past their humble beginnings as core temperature sensors in the 1960s with clunky single-marker setups.

With recent advances in microelectronics, bioengineering, mechanical engineering, and materials science, ingestible sensors that now integrate multiple functions (pH, pressure, temperature, optical image taking, etc.) into a swallowable capsule have become commercialized and mainstream (e.g., PillCam, VitalSense, myTemp) [59,60]. New studies demonstrate startling possibilities with capsule-based biopsies, surgical interventions, drug adherence, and drug administration becoming ever-closer to an available medical product [71]. Yet, there are still many technical challenges and design features specific to crafting a reliable IBC, and this section in particular focuses on device power, locomotion, localization, and safety.

### 3.1. Desired Features and Technical Challenges

**Figure 3:**
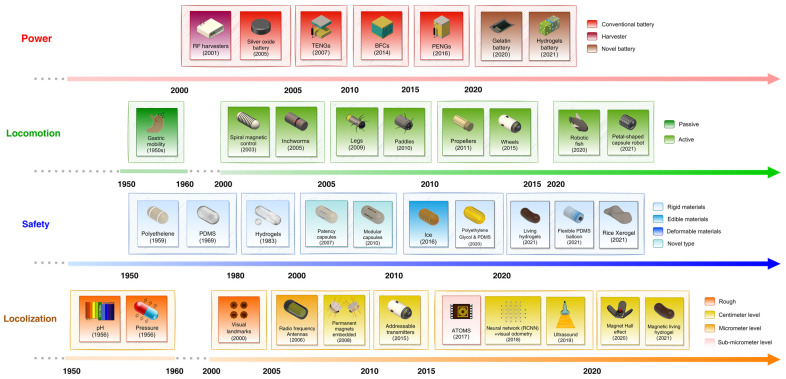
Scheme of IBCs design features: power [81,82,83], locomotion [84,85,86], localization [87,88,89,90,91,92,93,94,95], and safety [51,87,96,97,98,99,100] and levels of development over time. (Created with BioRender.com).

When first approaching IBCs, the notable challenge of powering IBCs comes to mind. While many commercial IBCs adopt a conventional zinc-silver-oxide or lithium-ion button cell approach, a plethora of novel IBC power sources have sprung up as alternatives since traditional button cell batteries still pose a risk when IBC shells unexpectedly break down or leak. Whether they be powered by gastric fluids [72,73,74], mechanical vibrations/deformations [74], stainless steel springs [75,76], or exterior magnetic fields [77,78,79,80], many interesting alternatives exist for the now standard button cell electronics approach, which are introduced in detail in Section 5. This section will mainly focus on IBC locomotion, localization, and of course, safety (Figure 3).

#### 3.1.1. Locomotion of IBCs

IBCs fall quite neatly into two categories in regard to locomotion: passive and active devices. Passive devices refer to IBCs that follow the GI tract’s peristaltic motion, acting as drifting sensors that follow the stomach’s “current”. These devices make up almost all of the commercial IBCs whether they be for sensing physiological markers, e.g., pH and gas, or for capsule endoscopy [71]. With passive devices, the uncontrollable nature of device locomotion creates a design challenge for many applications. Endoscopy capsules approach this problem by utilizing multi-camera systems, ultrawide angle cameras, and variable capture rates based on capsule velocity, ensuring adequate coverage of the target [99]. This kind of brute force approach extends to other passive IBCs that increase sampling rate to ensure more even data acquisition. However, recent advancements in IBCs have brought about a novel solution, namely active devices.

These devices can be magnet embedded, allowing for external control using magnetic fields created through permanent magnets and/or coils [77,78]. However, magnetically controlled devices carry certain risks and limitations, such as large forces being applied to surrounding tissues or IBCs potentially getting stuck in collapsed segments of the GI tract (e.g., intestines). Another approach to active IBCs integrated locomotion systems directly onto devices. Whether it be spider-like “legs” that anchor devices to intestinal walls, corkscrew like propeller systems, inch-worm like crawling segments, or miniature “paddles”, these active devices present their own unique risks that must be weighed with the benefits of steerable controllable IBCs. These devices, despite in vivo animal trials that demonstrate effectiveness, have high risks to cause further strain and damage to tissues in a potentially fragile GI tract. Finally, devices that combine both external magnetic and on-device locomotion systems have also been developed. These devices utilize external locomotion a majority of the time and only use on-device locomotion systems in a pinch [71]. Furthermore, the integration of these locomotion systems with essential sensors and therapeutic instruments are most certainly bottlenecked by current battery cell technology due to the relatively large power draw of electromechanical actuators.

#### 3.1.2. Localization of IBCs

With the vast expanse that is the 9-meter-long GI tract, capsules need to be effectively located to perform effective therapy or capture any notable data. Crude approximations of capsule locations can be performed with pH observations, oxygen concentration [100], or visual landmarks [99], allowing for a general understanding of gastrointestinal motility and rough monitoring of transit times in different sections of the GI tract. For more precise devices, such as lesion removal [79,80], focused monitoring [101], ulcer treatment [76], and other targeted therapy [77] capsules, significantly more accurate and precise localization techniques are critical. To achieve a more precise localization, IBCs can either use more advanced imaging techniques or vector position calculations based on radio frequency signals transmitted to external receivers in order to achieve centimeter level error margins. For an even more precise localization, permanent magnets embedded onto capsules can be sensed by external arrays of magnetic sensors in order to achieve millimeter scale accuracy [71]. For applications requiring further precision, addressable transmitters operated as magnetic spins are a novel approach allowing for MRI like precision without using superconducting magnets. Based on an in vivo study of mice, this localization strategy resulted in an error less than 500 µm [90]. Beyond localization, odometry can also applied to measure distances traveled within the GI tract (OdoCapsule) [101]. Currently, an IBC capable of measuring distances within the small intestine has been demonstrated, with three retractable leg-wheels that both act as odometers and keep the device orientated correctly.

#### 3.1.3. Safety Challenge of IBCs

Safety for IBC devices in split into two main barriers: one, device encapsulation and two, device retention. While encapsulation is an obvious enough technical challenge, device retention within the GI tract can result in blockages that may have lethal consequences.

First considering the main challenge of IBCs, numerous materials have been researched and investigated for their biocompatible nature in regard to encapsulation and adhesion (e.g., Parylene, PDMS, Polyethelyne, Biocompatible polycarbonates, plastics, and epoxies). While many IBCs adopt these materials as shells or encapsulation material, a majority of such materials are rigid and nondegradable, resulting in potentially lethal consequences if devices were to remain within the GI tract. Recent research into materials as well as novel IBCs forge a new path using edible, nutritive, and partially or fully degradable packaging materials and components [78,99,102]. From materials such as edible inert metals, trace elements below the RDI (recommended daily intake), their oxides, and nutritive organics, such as bio-pigments and polymers that are dissolved, digested, and absorbed after fulfilling their function (e.g., shellac coating). Furthermore, increased optimization of electronic circuit components to utilize non-battery powering mechanisms (e.g., gastric fluid, external magnetic fields, or physical motion) enable more conventional encapsulation materials such as traditional pharmaceutical tablet powders [74] or simply freezing functional aspects of the IBC into ice pills (as is the case with a surgical intervention IBC [78]).

Second, when devices overstay their welcome within the GI tract, capsule retention becomes a major health concern, especially so with passive sensing capsules. Despite capsule retention in patients being at 1.4%, many factors such as gut health, motility, and IBC size can significantly affect retention rates [103]. As of now, a multitude of approaches have been taken to combat this challenge. Looking to the commercial sector, PillCam has developed a patency capsule that mirrors their PillCam’s dimensions, effectively acting as a dummy which can self-dissolve in case the IBC remains within the GI tract [104]. By taking this before an IBC endoscopy procedure, healthcare professionals and patients alike can better understand their personal risk of capsule retention. Following the rapid maturing of this product category, many healthcare companies have fashioned their own capsule endoscopy systems, such as Jinshan Science & Technology’s competitively priced OMOM capsule [105]. Looking towards new research into novel IBC materials, new approaches that adopt deformable softer materials and dissolvable components can reduce retention and ease blockages that may occur. Furthermore, modular compartmentalized capsules that break down into smaller parts upon retention or engineering physically smaller IBCs due to miniaturizations in sensors, batteries, and/or electronics are all possible approaches to reducing unwanted GI tract retention rates.

Finally, the safety of IBCs with active robotic components should also be evaluated extensively prior to in vivo trials. With the advent of drug administration, surgical intervention, and biopsy IBCs, rigid components directly interfacing with surrounding tissue may cause extensive physical strain, perforations, or even scarring on GI tract walls. As is the case with passive IBC retention, flexible, soft, and dissolvable materials should be favored.

### 3.2. Detectable Indicators of Physical Health

Ingestible sensors can monitor a wide range of indicators, such as the optical appearance of tissue, local biomarkers (such as electrolytes, metabolites, and enzymes), the microbiome within a patient’s GI tract [106], and various physical indicators (e.g., pH, pressure, and temperature). This enables ingestible sensing technology to be applied in tissue imaging, monitoring of gastrointestinal inflammation, health status, and motility among a plethora of other applications. Furthermore, IBCs have the ability to assess medication compliance [74] and even deliver on-demand or extended-release medication to targeted areas [71,77,106,107]. The first generation of ingestible electronics were demonstrated in clinical proof-of-concept studies that they could be used to measure pressure, temperature and pH in the GI tract. With further advances in numerous fields from microelectronics to bioengineering, ingestible biosensors have now expanded to fulfill a variety of roles within the GI tract, expanding beyond simple diagnosis and tracking to perform surgical intervention [78,79] and drug administration [71,77,79,108]. In Table 3, we focus on the various types of IBCs with applications in the stomach, intestines, and GI tract as well as the markers sensed by these capsules. While these capsules can be applied throughout the entire GI tract, this table presents the main usage scenarios for these capsules, and with the challenging environment that is the GI tract, special attention is paid towards each IBCs’ encapsulation material.

#### 3.2.1. Sensing Devices

The primary branch of IBCs in development ever since ingestible core temperature sensors of the 60s are still sensing devices. These IBCs seek to tap into the vast sea of physiological and biochemical markers present within the GI tract, hoping to make sense of the physically, chemically, and biologically complicated environment that is the gut’s microbiome. While conventional devices that measure physiological indicators. such as pH, pressure, and temperature, have already made leaps and bounds that result in commercial products patients and health care professionals can utilize today [114], many novel devices have been developed which enable monitoring of the gaseous chemical makeup of GI tract [100] or the biomarkers indicative of certain diseases and/or medical conditions [105]. Furthermore, advancements in micro-scale camera technology have led to the widespread adoption of endoscopy IBCs, such as Medtronic’s PillCam systems, as opposed to more invasive and painful traditional endoscopy methods [99,104]. However, these systems require patients to fast prior to IBC consumption in order to ensure a “clean” GI tract. Furthermore, they fail to provide the necessary clarity at times—resulting in the development of novel sensing technologies from ultrasound imaging & various other forms of EM spectroscopy to odometry IBCs that enable stabilized video/photo capture [71,112,114,115]. Yet, while sensing IBCs are able to access, monitor, and provide a whole host of important diagnostic data to healthcare professionals about patient health, an entirely new type of IBCs can potentially revolutionize how the healthcare industry treats those diagnosed with GI tract diseases/conditions.

#### 3.2.2. Operational Devices

Another branch of IBCs that has recently developed are IBCs which carry out complex operations within the GI tract. These operations include biopsies, surgical interventions, and drug administration. IBCs which carry out biopsies primarily function through magnetically controlled nested razor setups, allowing for a non-harmful amount of GI tract tissue to be removed & stored within the capsule [79,80,110]. This considerably less invasive manner of collecting tissue from the GI tract enables tremendously more targeted and location-specific biopsies. However, these devices are still in developmental phases with only prototype devices being built. Chugging further along their developmental cycle are IBCs for surgical interventions. These devices can carry out a plethora of functions such as patching stomach ulcers while delivering pharmaceuticals to wounded areas [77] or removing foreign bodies (such as accidentally ingested button cell batteries) [78]. Achieving such complex functions require significant advancements in mechanical engineering, a feat reflected in the adoption of novel folding methods inspired by origami within device designs, and similar to biopsy IBCs, surgical IBCs tend towards magnetic forms of localization and locomotion due to their structural nature preventing use of conventional battery cells. These surgical IBCs have successfully moved past prototype devices and gearing up for testing in animal trials. Finally, IBCs for drug administration, having been successfully tested in animal trials, prove to be a direction of significant interest, delivering drugs to targeted locations on demand [71,76,106,108,116,117]. Adopting more conventional power methods alongside novel ones (such as chemically inflatable “needles” [107]) as well as novel approaches to locomotion (such as self-orienting capsules [75], administrative IBCs progress towards a future of painless, targeted medicine to soothe gastrointestinal diseases. Furthermore, extended-release administrative capsules can release drugs as necessary, remaining in the body over a month to release drugs [106]. However, IBCs must still solve the sizing restraints that come with an ingestible platform as, ultimately, the drug dosage is still bottlenecked by IBC sizes.

## 4. Implantable Biosensors for Healthcare

With the developments of novel device structures, advances in flexible and compatible materials (such as the biodegradable materials, biocompatible hydrogels and conductive nanocomposites [118]), and the work to reduce the biological reactions of sensors, the research and applications of the implantable biosensors are proposed and improved to realize high quality detection and monitoring in vivo which are capable of physiological and electrical examinations in the real-time diagnosis or long-term sensing for targeted treatments.

### 4.1. Challenges and Features of Implantable Biosensors

#### 4.1.1. Immune Biocompatibility

However, the complex response mechanism of blood and organs/tissue to foreign devices impede the progress and applications of the implantable biosensors [119]. According to the earlier literature, the body responses to the implantable biosensors mainly come from blood, subcutaneous tissue, and neural tissue [120,121], which may be similar to each other but not exactly the same. Sensors in vivo should minimize the injures to target organs/tissue, improve their performances, and achieve long-term biocompatibility, which puts immune biocompatibility an essential research focus. Besides, for future healthcare application of implantable biosensors, there is a great need for more in-depth understanding of immune response and a definition of exposure criteria under various circumstances, such as skin contact, intake, inhalation, and injection [19], as most of the design strategies introduced in the wearable and ingestible sensors are still applicable to implanted sensors, but with more stringent requirements.

On the one hand, once the biosensors are implanted into the blood environments, it is inevitable for almost all biosensors to cause protein adsorption, which will lead to the platelet adhesion and other subsequent biochemical cascade later [121]. Firstly, these phenomena cause a passive analyte diffusion barrier which decrease the sensibility and interfere the stability of the signals. Secondly, over time, the conformation of the surface adhered proteins and the platelet further adhere to form a thrombus which does harm to the health of the patients. On the other hand, as for the responses of subcutaneous tissue and neural tissue, the implantable biosensors arouse severe inflammatory and the foreign body response (FBR) which absolutely leads to the loss of sensibility and reliability. Specially, different from subcutaneous tissue, the implantable biosensors bring about a breach of the blood–brain barrier and damage to the underlying neural tissue [122]. Besides, the existence of nervous system-resident astrocytes and microglia in neural tissue contributes to the formation of a glial scar in FBR [123].

In order to restrain the platelet adhesion and the form of a thrombus, Soto et al. reviewed the strategies, which can be divided into four methods, namely the use of hydrophilic and zwitterionic materials, controlling identity and conformation of adsorbed proteins, heparin immobilization, and the use of heparin-mimicking materials, nitric oxide release. As for the strategies to curb the responses from subcutaneous tissue and neural tissue, we can use zwitterionic materials or porous and nanopatterned coating materials. Moreover, the release of tyrosine kinase inhibitors or dexamethasone or nitric oxide work [124].

#### 4.1.2. Other Desirable Features

Facing the complex and changeable microenvironment, which consists of the mixture of multiple elements and chemicals, the rapid development of the implantable biosensors focuses on solving the essential issues that improving the key performances as the selectivity. In general, along with the immune biocompatibility and selectivity, the implantable biosensors still have a long way to go to improve the performance of the limit of detection, sensitivity, sensing reliability, sensors long-term compatibility, and flexibility in vivo.

### 4.2. Detectable Indicators of Physical Health

The implantable biosensors have more complicated targets and indicators as they have direct contact with blood and tissue when they are implanted into human body [91]. We summarize the indicators of implantable biosensors in Table 4. As an important part of implantable biosensors, we list the mechanical pressure part separately, which can be divided into intracranial pressure, intraocular pressure, pressure in artery, intra-abdominal pressure, and intra-bladder pressure.

#### 4.2.1. Physiological Signal of Implantable Biosensors

Currently, implantable biosensors are more comprehensively investigated for neuromonitoring, which can detect electrophysiological signals spontaneously generated by nerves (e.g., action potentials, etc.) or electrical pacing signals generated by implantable devices (e.g., stimulation of the pallidum by DBS, etc.), thus promising for monitoring and early warning of neurodegenerative diseases (e.g., Parkinson’s disease), diseases with abnormal brain electrophysiology (e.g., epilepsy), and psychiatric disorders (e.g., schizophrenia), and for evaluating the efficacy of surgical electrical stimulation.

However, implantable neuromonitoring sensors face challenges in numerous aspects. On the one hand, in terms of construction, electrode size, excessive electrode spacing, and insufficient electrodes lead to inaccurate recordings [139]. On the other hand, insufficiently flexible materials prevent the device from adapting to the curved surfaces of the brain. Therefore, new structures and biocompatible materials or new innovative sensor devices have been invented. An enhanced-mode, internal ion-gated organic electrochemical transistor (e-IGT) based on reversible redox reactions and a reservoir of hydrated ions within a conducting polymer channel has been developed, which enables chronic intracranial brain imaging from the brain surface, deeper structures in freely moving rats, and even real-time detection of epileptic discharges [140]. Furthermore, a neural fringe with ultra-small size and high flexibility compared to conventional structures is proposed, consisting of flexible and high aspect ratio microelectrode filament arrays that can stably record electrical signals and neural activity [125]. Notably, deep brain stimulation (DBS) is a widely used method for the treatment of neurological disorders. A fully implantable device with both chronic electrophysiological recording and stimulation has been shown to be effective. The device possesses high resolution, low noise, and stimulation artifacts [125]. In addition, a CMOS 256-pixel photovoltaic-powered implantable chip with an active pixel sensor has been invented that enables visual imaging based on electroretinogram (ERG) measurements [130].

#### 4.2.2. Mechanical Pressure of Implantable Biosensors

Combining the superiorities of light weight, high sensitivity, and low cost, flexible pressure sensors based on the mechanism of piezoresistive effect, capacitance, and piezoelectricity represent an essential part of flexible electronics. Firstly, as intracranial hypertension is a condition possibly caused by traumatic brain injury, aneurysms, brain tumors, hydrocephalus, stroke, and meningitis, for monitoring, an implantable pressure sensor is fabricated to continuously and wirelessly monitor intracranial pressure (ICP) [135]. It is worth mentioning that Chen et al. report implantable and bioabsorbable multifunctional sensors for the brain, which can continuously monitor ICP and at the same time minimize the risk of infection and reduce the pain for patient. Secondly, the accurate and continuous elevated intraocular pressure (IOP) monitoring can prevent or relieve the decreased vision and blindness. A multifunctional contact lens sensor with sandwich structure is reported to continuously and wirelessly monitor IOP. Thirdly, considering the significance of blood flow detection for recovery after surgeries which is used to assess the status of vascular bed, a pressure sensor which consists of a bilayer coil structure for radio frequency data transmission and a fringe-field capacitive pressure sensor is reported [136]. Fourthly, high intra-abdominal pressure (IAP) is associated with acute renal failure and lung injury, which is called abdominal compartment syndrome (ACS), causing high morbidity and mortality. A set of IAP monitoring systems were used to record IAP data and analyze the relevance among stressors in the body, leading to diagnosis and medical treatment [137]. Lastly, intra-bladder pressure (IBP) is related to diabetes, aging, neurologic diseases, and even the ACS mentioned above which leads the underactive bladder (UAB) syndrome. A self-control system consisting of a triboelectric nanogenerator sensor and the actuator to realize the autonomous micturition and monitor the fullness of the bladder and IBP was introduced [138].

#### 4.2.3. Biochemicals of Implantable Biosensors

The presence of biomolecules in tissue fluids or other body fluids reflects the regulation of metabolic processes or physiological homeostasis in the human body, and reliable monitoring of their composition and concentration not only provides an assessment of the body’s status, but also aids in the real-time kinetic observation of biomolecules and chemicals in vivo. However, the coexistence of a large number of substances with similar structures and properties in a variable and complex micro-physiological environment poses a challenge to the sensing selectivity and reliability of implantable biosensors.

Currently, the in-situ detection of important substances, such as dopamine (DA), hydrogen ions, and glucose, has received increasing attention. As an important neurotransmitter, DA is widely distributed in the central and peripheral nervous system and is associated with numerous functions such as cognition, movement, and emotion in humans. Therefore, studies on the detection of DA have shown its importance in physiological and clinical applications. Based on the basic carbon fiber electrode structure (CFE), it is proposed that CFE coated by PEDOT/graphene oxide can detect DA quickly with satisfactory sensitivity and exhibit a DA sensitivity of 880 ± 88% without significantly altering the electrode kinetics [131]. Meanwhile, carbon fiber microelectrodes (CFMEs) modified with copper(I) sulfide functionalized graphene oxide nanocomposites (Cu_2_S/RGO) exhibited high selectivity for DA while avoiding interference from other components such as histidine, ascorbic acid, and uric acid [141]. At the ionic level, monitoring of central nervous system (CNS) pH in the living brain contributes to the understanding of acid-base and ion homeostasis on brain activity and effects. Hao et al. demonstrated a potentiometric method for monitoring CNS pH in vivo with carbon-fiber-based proton-selective electrodes (CF-H^+^ISEs), which is highly resistant to fouling and can disclose and explain that brain acidosis is caused by CO_2_ inhalation and brain alkalosis is caused by bicarbonate injection [134]. In addition, continuous monitoring of pH in the living brain may also provide and facilitate the detection and monitoring of positive ions, such as K^+^, Na^+^, and Ca^2+^, in vivo. A novel K^+^-sensitive miniature solid-state ion-selective electrode probe, based on a PEDOT electrode sheet, was fabricated to measure multiple parameters associated with the neurological phenomenon spreading depression (SD), which is thought to be important in brain disorders, such as stroke, traumatic brain injury, and migraine with aura [64].

In addition to the detection of substances in the brain environment, glucose concentration in the blood is an important biochemical parameter to assess the level of control of diabetes, and continuous glucose monitoring also helps to understand the glycemic index (GI) of food to the individual with the regulation of the frequency and dose of medicinal insulin. A flexible enzyme electrode sensor with a cylindrical working electrode modified with three-dimensional nanostructures was creatively implemented for glucose monitoring based on interstitial fluid (ISF) analysis by rotational inkjet printing technology and solved the problem of sensing sensitivity for monitoring even under hypoglycemic conditions [132]. Moreover, based on the concept of artificial organs, functional implantable glucose biofuel cells have also been applied to monitor blood glucose [142], which enlightens us to understand and design sensors at the level of living sensory cells or organs.

## 5. Strategies for Reliable Biosensors

Nowadays, whether in the precaution of chronic diseases, clinic recovery and treatment, or the monitoring of vital signals in daily life, people’s need for long-term monitoring of reliable biosensors, which can realize continuous and stable detection and provide a large amount of valuable data, has sharply increased. Besides, some ingestible and implantable biosensors introduced above need to be replaced periodically which causes both mental and physical suffering to patients and increases the medical cost and risks too.

Hence, for the realization of reliable biosensors, scholars have made lots of efforts and attempts. In many cases, the monitoring duration of these biosensors largely depends on the energy provided to the devices or the time to recharge, assuming the devices do not fail as the same time. Meanwhile, ingestible biosensors and implantable biosensors detect signals in vivo under more complex conditions and cannot have direct connection to the outside environment, which make the power supply and management an essential issue worthy of being considered. In addition, we can also design from the prospective of structures and materials of the biosensors to improve their reliability.

### 5.1. Strategies to Improve Reliability of Biosensors

Biocompatibility which refers to materials that cause appropriate reactions in specific parts of the body [143] is a major issue to the application of sensors. According to the interpretation of the International Standards Organization (ISO) meeting, biocompatibility refers to the ability of living organisms to react to inactive materials, and generally refers to the compatibility between the material and the host [144]. Meanwhile, the manifestations of biocompatibility vary by the types of the biosensors according to the context above as the wearable biosensors address on the mechanical biocompatibility, the ingestible biosensors focus on the encapsulation and the implantable biosensors attach the importance to the immune biocompatibility. In short, we can mainly think about the ameliorating of materials and structures to promise the high reliability of the biosensors. As a representative of glucose biosensors, materials like transition metal oxides, e.g., Co_3_O_4_, TiO_2_, CuO, and NiO, exhibit better stability than the metals, e.g., gold, platinum, and their alloys [145]. Besides, with the wide use of nanomaterials, e.g., metal nanoparticles, conductive nanotubes, silicon nanowires, and polymer materials, such as the PDMS, PI, and PET promotes, the performance improvement of biosensors helps to prolong their monitoring time. As for biofuel cells using as power source for biosensors, an electrode with a 3D structure can improve the stability of the immobilization of enzymes which enhanced the reliability from the structure. There are also many other methods and strategies in engineering prospective to prolong the monitoring time of the biosensors which means we will always have the chance to explore new field to realize the long-term monitoring indeed.

### 5.2. Energy Sources and Power Management of Biosensors

The great limitation for the biosensors to carry out their functionalities and realize long-term monitoring is the indemnification of a continuous power supply. We summarized the power sources that are applied to the wearable, ingestible, and implantable biosensors, including the outer power supplies and the advanced self-powered sources. According to the Table 5, the power sources include batteries, photovoltaic (PV) or solar cells, radio frequency (RF) energy harvesters, biofuel cells (BFCs), and energy generators consisting of piezoelectric nanogenerators (PENGs), triboelectric nanogenerators (TENGs), electromagnetic generators (EMGs), electrostatics generators (ESGs), and thermoelectric generators (TEGs) which are able to maximally collect the energy from the ambient environment and human body for use. There are also hybrid energy strategies to expand the power capacity and improve their performance which are widely used in wearable biosensors.

As batteries with simple structure provide power without the favor of complex circuit, they are comprehensive used as the power supply for biosensors at the very beginning. When it comes to batteries, the lithium-based batteries, e.g., lithium batteries and lithium-ion batteries, which are both made of lithium, possess high energy density and battery voltage promoting their widespread use in biosensors and medical applications [191]. Considering the safety problem and high cost, the rechargeable batteries based on the alkali metals and alkaline earth metals are proposed as the substitute for the lithium-based batteries. For the wearable biosensors, all kinds of batteries, such as alkaline batteries, nickel-based batteries, and lithium-ion batteries, are used as the power to measure temperature or detect the lactate, pH, and ions from the body fluid on the body surface. The implantable biosensors adopted the lithium-based batteries as the energy resources to realize the information collecting of heart beating and neural signals. Meanwhile, for ingestible biosensors, silver oxide batteries consisting of silver oxide as the cathode and zinc as the anode have edges over other batteries as the power supply because of their safety and the refrain of the thermal runaway. In order to further improve the compatibility and the performance of the batteries in terms of weight and volume as well as the toxicity of the batteries, which presents inconvenience and suffering to people, restricting their applications, various batteries like the solid-state batteries and transient batteries are being developed rapidly. For instance, Young et al. demonstrated a kind of biodegradable aqueous sodium-ion energy storage device for the ingestible biosensors to achieve power supply for medical signals sensing and avoid the toxicity problem as the same time [192]. PV/solar cells with the characteristics of light weight, high flexibility and efficiency have aroused great interest from scholars and become ideal substitutes for batteries. As a power supply for sensors, there are self-power pressure and stain wearable sensors with solar cell utilizing the ambient light as the power source to realize continuously and stably measurement [154,155]. Besides, a photovoltaics-powered chip mentioned above was proposed for implantable active pixel sensors to detect the optical signals [130]. RF harvesters as continuous and controllable power sources are able to collect the RF waves energy from the dedicated or ambient environment. A textile-based large area RF-harvesting system was introduced, and it demonstrated potential application for wearable sensors. They pointed out their potential value in defense, space, smart home, and childcare for sleep monitoring and location tracking [155]. Moreover, it is worth mentioning that the transcutaneous energy transferring devices are needed for ingestible and implantable biosensors to harvest energy compared to PV/solar cell or RF harvesters applied in wearable biosensors.

A biofuel cell generates energy by redox reactions between the anodic site and the cathodic site which can produce continuous power for long-term monitoring as the reactant are present. The most notable feature of biofuel cells is their ability to make use of the reactants available from the human body. In different kinds of biosensors, the biofuel cells can extract energy from various body fluids, e.g., sweat and tears, via wearable biosensors, gastric and intestinal juice via ingestible biosensors, tissue or cell fluid and blood via implantable biosensors, and for electronics to detect the glucose, lactic acid, medication adherence, temperature, humidity, and so on. Meanwhile, the low power conversion efficiency and the dependence of the reactant limit their applied range and area.

According to different electricity generation mechanisms, here, the power source generators can be divided into five types as piezoelectric nanogenerators (PENGs), triboelectric nanogenerators (TENGs), electromagnetic generators (EMGs), electrostatics generators (ESGs), and thermoelectric generators (TEGs). The five kinds of generators or nanogenerators can utilize material characteristics or energy transducers to transfer deformation or thermal energy to power. Based on the forming principle of electric dipole in piezoelectric materials when the force is applied, PENGs can generate the voltage difference to power devices to monitoring the signals of pressure, motion, breathing, heart beating, pulse and blood pressure. Based on the effects of friction electrification and electrostatic induction, till this moment TENGs can provide energy to wearable and implantable biosensors to detect the targets of tactile, breathing, pulse, motion, and blood pressure, and ESGs can be used as a power source for the vibration wearable sensor. Based on the generation of electromotion from the changes in the external magnetic flux of a close-loop circuit, EMGs harvest the kinetic energy and transfer it to power sensors to monitor the motion of wearable biosensors and detect the heart beating of implantable biosensors. Based on the pyroelectric effect, TEGs on the one hand can offer energy for wearable biosensors to monitor temperature, heartbeat, SpO_2_, and body acceleration, and on the other hand, make use of the heat energy in the intestines and stomach of the human body to power ingestible biosensors to detect temperature, pH, and iron ions. In order to improve the performance of the energy sources by increasing the PCE and the stability, researchers have tried and realized several kinds of combinations of the energy sources mentioned above, including the combination of PENGs and TENGs, PENGs and TEGs, PV and TEGs, TENGs and solar cell.

## 6. Transforming Healthcare Technologies with Biocompatible Biosensors

Over the decades, researchers have developed a variety of compelling biosensors, some of which are already commercially available. These biologically compatible sensors are realizing indoor-feasible detection and revolutionizing current healthcare technologies. From the perspective of application level, the new generation of healthcare technology presents three forms: the next generation of intelligent diagnosis, integrated diagnosis and treatment equipment, and the improvement of medical service and management level (intelligent bedside care, chronic disease management, and improvement of inpatient treatment efficiency in specific).

### 6.1. A Prototype for the Next Generation Diagnostics

Currently, in most healthcare facilities, tests are still prescribed by physicians, which in turn are performed by laboratories and reports are issued for physician diagnosis and ultimately collected in the form of electronic or paper-based medical records. This model allows clinical practices to generate rich data, but they are still not systematically integrated and analyzed using specialized data analysis methods.

**Figure 4:**
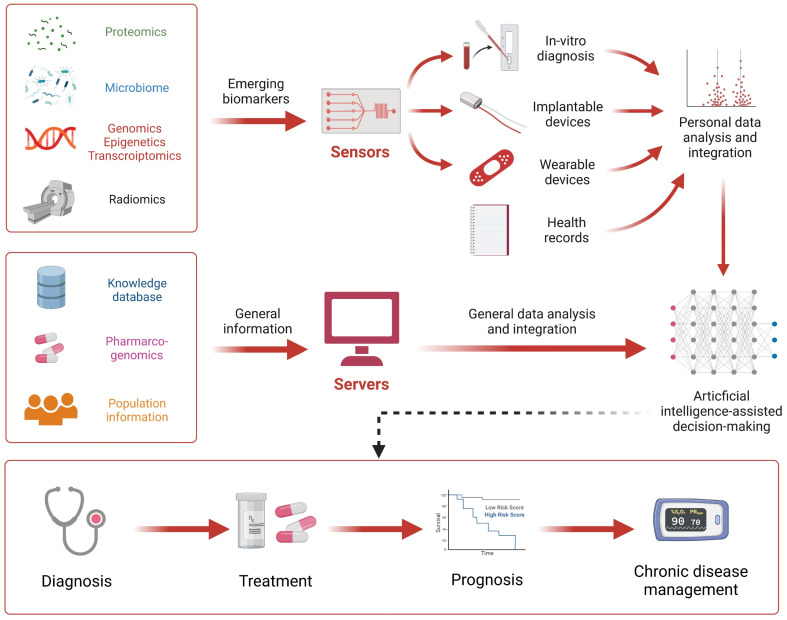
Scheme of a prototype for the next-generation diagnostics. (Created with Biorender).

The core connotation of next-generation diagnostics is the personalized diagnosis and precision medicine. Although various traditional sensors and biocompatible sensors provide multi-dimensional data support for the development of precision medicine, it is still essential to apply the generated data. To overcome the barriers of data acquisition and data analysis, intelligent algorithms have been introduced into medical sensing systems, greatly facilitating the development of personalized precision diagnosis (Figure 4). The organic combination of these novel biocompatible sensors and artificial intelligence (AI) is considered to be the prototype of next-generation diagnostic technologies. Artificial intelligence algorithms can significantly improve diagnostic accuracy and maximize the physiological signals in situ. For example, Veeralingam et al. reported the first nanomaterial-based multifunctional sensing platform for simultaneous and continuous monitoring of specific important body parameters, namely skin hydration levels, glucose concentration, and pH of biofluid sweat, with high accuracy and speed [193]. To facilitate a human–machine interface capable of analyzing large sample sizes of data, the sensor is connected to an open-source microcontroller board (QueSSence) in which an AI-based K-nearest neighbor (KNN) algorithm is capable of acquiring data accurately and quickly from complex mathematical nodes.

Notably, AI algorithms and models bridge the gap for integrated and comprehensive sensing and health assessment based on environmental sensors and biocompatible sensors. This prospective concept has been tested on animals. A wearable multi-sensor system was explicitly designed by Zhang et al. to continuously obtain real-time data on environmental and physiological parameters of live sheep [194]. Predictive models for comfort and health evaluation were developed based on generalized regression neural networks (GRNN) for environmental and physiological parameters and backpropagation neural networks (BPNN). The results show that the wearable multi-sensor system has high accuracy and stability in data acquisition, and its power consumption and communication performance can meet the monitoring requirements. Importantly, correlation analysis showed a significant correlation between environmental and physiological parameters. Thus, in the case of unknown essential health parameters, only environmental information is available to predict health levels. This inspires us to predict the health status even by changes in environmental parameters, and the assessment of human health should include the assessment of the environment, which is the connotation of Ambient Intelligence [195]. At this level, the algorithm also provides a method for the fusion of sensor data, including SVM, Bayesian algorithm, Kalman method, K-means algorithm, convolutional network, and other algorithms that provide many sensor data fusion solutions, helping to achieve comprehensive functions, such as emotion and motion pattern recognition (Table 6).

### 6.2. Integrated Systems for Therapeutic Interventions

Precision therapeutic interventions, including but not limited to exercise rehabilitation [6] and drug therapy [217], are important to achieve personalized medicine, where the sensors could provide information from variable modalities. For example, the Kinesia system, developed by GLN, monitors Parkinson’s disease through a patient-worn sensor and tablet-based software. The tablet is responsible for issuing instructions to the patient, collecting data from the sensor, and transmitting the data to a cloud service. Through a portal, physicians can access information about the patient’s progress in recovery. It can be used to monitor the progress of the disease and evaluate the effectiveness of treatment for Parkinson’s patients participating in clinical trials, as well as to help neurologists adjust the settings of implanted brain pacemakers (deep brain stimulation, DBS). The system’s sensors capture linear acceleration and angular velocity, which are then processed and converted into component values by GLN software, allowing physicians to determine the severity of the tremor over a fixed period of time.

**Figure 5:**
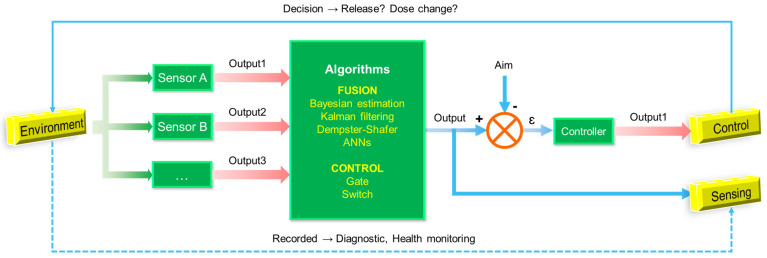
Scheme of a prototype for the next generation diagnostics.

From this level, micro or macro cybernetic design is particularly important, as shown in Figure 5. While the sensor performs the sensing task, the information it senses needs to be used as a reference for immediate actions or long-term decisions to reflect the system’s grasp of environmental information. From this perspective, there is a slight lack of current research. This is partly due to the fact that most of the current sensor research lacks systematic design, and the reliability of its sensing results, although high, may not provide a practical or perfect IO interface. Therefore, subsequent research needs to strengthen the design of wireless communication and upper computer processing to algorithmically realize the upper computer mining and fusion of sensor information. On the other hand, how to establish a processing system and decision system corresponding to the signals collected by the long-range sensors still needs to be determined by actual experiments with a large sample of people.

### 6.3. Improvement of Medical Services and Management

While healthcare services and management have been initially computerized, bedside care and healthcare management are advancing toward intelligence through the integration of biocompatible sensors and sophisticated environmental sensors. Internet of Things (IoT) technology has greatly facilitated the development of clinical management and telemedicine. While biocompatible sensors can detect more information in situ, the accessibility and transferability of information are equally important for applications, especially for ingestible sensors that function in the digestive tract and implantable sensors that are implanted in the human body. Therefore, it is necessary for biocompatible sensor terminals to communicate with cloud or relay devices represented by cell phones via far-field radio frequency (RF) communication, skin electrodes, wireless networks, Bluetooth, etc.

The use of this communication will bring many benefits. First, an aging society is accompanied by a higher incidence of chronic diseases and the consequent burden of high medical bills. Sensors provide physicians with the ability to remotely monitor patients’ body parameters, helping to enable telemedicine and personalized treatment while reducing the strain on the healthcare system and minimizing its operational costs. Second, a prominent issue in clinical care is medication adherence. For example, in the treatment of tuberculosis, the treatment strategy is to encourage patients to adhere to the treatment regimen, and one of the measures is directly observed therapy (DOT), in which the staff supervises the patient in a direct interview to take anti-tuberculosis drugs. Moreover, non-adherence to chronic medication regimens can lead to delays in treatment and a drain on health care resources. It also imposes a preventable financial burden of hundreds of billions of dollars on the government. To address this issue, ingestible electronic devices are ideal and can be included in conventional medications to address medication non-adherence. Oral medications can be tagged with a radio frequency identification (RFID) chip, such as the Proteus Discover, which monitors patient adherence to a specific treatment regimen [218]. After ingestion and contact with gastric juice, the Proteus Discover system is powered by an electrical couple and communicates its identification code to a receiver patch worn by the patient. A proof-of-concept study to assess transmission efficacy has been conducted by attaching the chip to an inert pill to be taken with TB medication.

## 7. Concluding Remarks and Prospects

In the past decade, biocompatible biosensors have been able to access a wide range of physiological health information. In situ, real-time, simultaneous multi-marker detection has provided a richer resource or information modality for precision medicine and personalized diagnosis. Advances in materials science, device design, and processing methods have made long-term, real-time monitoring possible, helping to monitor the progression of chronic disease, understand its course, and further identify potential biomarkers.

However, the application and research of biocompatible sensing technologies in the medical field is still limited, especially in chronic disease care and invasive, implantable sensing applications. Most of the research has focused on addressing the crux of biosensors and improving their key properties, such as sensitivity, selectivity, reliability, and compatibility. Furthermore, most of the current research on intracellular monitoring has only demonstrated the feasibility of its principles and functions, and this area is still worth exploring.

Furthermore, to apply biosensors in clinical practice, their use must be considered for people with quality-of-life requirements. Therefore, the sensing technology should maximize the quality of life of the people it is used on to achieve the best detection results and should also be affordable for the patients. Commercialized wearable electronics and IBCs provide a good reference for the next technological development. On the one hand, biosensors should be progressively disposable, especially for ingestible and implantable biosensors, or for wearable biosensors that can be easily recycled, which brings convenience to patients and raises the requirements for long-term monitoring and low-cost biosensors. On the other hand, biosensors should minimize the physical sensation they cause to users, including improving flexibility and reducing size, while new synthetic materials and novel structures can be used as well as the development of three-dimensional packaging and integration technologies to facilitate the miniaturization process, making biosensors more portable and lightweight, all research directions worthy of deeper exploration. Taking electronic skin as an example, electronic skin is self-powered by solving the power supply problem with nano-generators, constructing arrays using multiple single electrodes and multi-axis sensors, and gradually achieving miniaturization through a strategy of integrating sensor devices, wireless units, and systems. Moreover, miniaturization comes at the cost of shrinking in situ user interfaces. Thus, the ergonomic and user-friendly design of user interfaces with wireless devices is important, which will improve user experience and reduce their barriers to carry and use biosensors, which puts more demanding requirements on industrial design.

Currently, researchers have fully elaborated various sensing technologies in a number of common disease application scenarios and have made some useful explorations in AI algorithm-assisted precision diagnosis and improved healthcare service management. As electronic devices are integrated with biological bodies, future challenges are increasingly apparent, e.g., in terms of ethics, security, communication, and control. Sensing technologies suitable for application scenarios should be carefully selected, and issues, such as the processing of massive multimodal data, information fusion technology, and integrated management of multi-individual data, should be fully considered, where user privacy protection and ethical issues will be addressed throughout. We should further consider ethical and emotional issues based on a full consideration of biocompatibility issues to achieve more comprehensive compatibility between devices, users, and institutions.

## Figures and Tables

**Table 1:** Different modality of detection in wearable biosensor system.

Modalities			Sensing Elements/Sensor Types	Applications ^#^
Electrical	Cardiac (ECG)	Heart failure	Advanced skin-attachable electordes	●[13]
	Muscular (EMG)	Emotional valence	pre-gelled, self-adhesive Ag/AgCl electrodes with inter-electrode spacing [14]	●[14]
		Tremor	triaxial accelerometer, triaxial gyroscope, etc. [15]	●[15]
		Posture recognition	a surface EMG acquisition system with EMG acquisition circuit and an MCU with ping-pong buffer designed clock sources	●[16]
	Cerebral (EEG)	Seizure	Subcutaneous devices: 24/7 EEG SubQ™, Minder^®^, etc.	●●[17]
			Surface devices: Behind-the-ear EEG, e-Glasses, Ear-EEG, Sensor dot, etc.	
Physical	Mechanical	Blood pressure/Pulse	Strain sensor or stretch sensor based on piezoelectric effect, piezoresistive effect, triboelectric effect	●●●[18,19,20,21,22]
		Motion	Strain sensor or stretch sensor based on piezoelectric effect, piezoresistive effect, triboelectric effect, optical fibers, myoelectric sensors	●●●[23,24,25,26]
	Acoustic	Human speech	Strain sensor or stretch sensor based on piezoelectric effect, piezoresistive effect, triboelectric effect; electret condenser microphones; acceleration sensors	●●[27,28,29]
		Cardiac/Respiratory sound	Optical sensors (bragg gratting), piezoelectric MEMS acoustic sensor, commercial microphone	●●[30,31,32,33]
	Temperature		Cr/Au metal microwires [34]	● [34]
Biochemical	In sweat	Glucose	glucose oxidase immobilized within a permeable film of the linear polysaccharide chitosan [34], Pt-decorated graphite or GOx/Pt-graphite [35]	●●[34,35]
		Lactase	lactate oxidase immobilized within a permeable film of the linear polysaccharide chitosan [34], a reference electrode for the lactate sensor with an internal copolymer of sulphonated polyesther ether sulphone–polyether sulphone (SPEES/PES) membrane as internal layer [36]	●[34,36]
		Na^+^	ion-selective electrodes (ISEs) [34,36,37]	●[34,36]●[37]
		Cl^−^	ion-selective electrodes (ISEs) [34]	●[34]
		K^+^	ion-selective electrodes (ISEs) [34]	●[34]
		pH (H^+^)	H^+^-selective PANI film that electrochemically deposited onto a Au electrode by cyclic voltammetry [38], Iridium oxide pH electrodes [36]	●[36]●[38]
		Uric acid	CF-reinforced electrode [39]	●[39]
		Cortisol·	MoS_2_ nanosheets dispersed within the pores of a porous polyamide (PA) membrane [40]	●●[40]
		Alcohol	Electrode casted by mixed droplet of AO_x_ enzyme, BSA stabilizer, and chitosan solution [41]	●[41]
		Ascorbic acid	CF-reinforced electrode [39]	●[39]
		Heavy metal (Hg^+^, Zn^2+^, Cd^2+^, Pb^2+^, Cu^2+^)	electrochemical square wave anodic stripping voltammetry (SWASV) on Au and Bi microelectrodes [42]	●●[42]
		Ca^2+^	ETH129 as the Ca^2+^-selective ionophore [38]	●[38]
		NH_4_^+^	ammonium-selective membrane containing nonactin, 2-nitrophenyl octyl ether (o-NPOE) and poly(vinyl chloride) [43]	●[43]
	In interstitial fluid (ISF)	Glucose	a screen-printed layer of Pt/C composite ink [44], Ag/AgCl electrodes [45]	●[44]
Multimodalities	ECG + lactase		ultrasonic transducers (HR and BP), electrochemical sensors (glucose in ISF and lactate, caffeine and alcohol in sweat) [11]	●[11]
	ECG + glucose		Self-assembly highly porous PEDOT:PSS hydrogel on paper fiber serving as a low-impedance ECG electrode and a glucose sensor	●[46]
			A hybrid skin patch with MEMS system	●[47]
	Ultrasonic + multiple biomarkers		ultrasonic transducers, electrochemical sensors [48]	●[48]
	sweat metabolites (such as glucose and lactate) + electrolytes (such as Na^+^ and K^+^) + skin temperature (to calibrate the response)		ultrasonic transducers, electrochemical sensors, and thermocouple [34]	●[34]

# Applications: ● For physical activity monitoring; ● For disease diagnostics; ● For chronic disease/routine habit monitoring; ● For researches.

**Table 3:** Summary of sensing and operational devices of IBCs.

Types	Feature	Encapsulation	Life Time	Development Stage	Applications	Year
(**A**) IBCs that located in stomach						
Drug Administration	Less painful, extended/on demand targeted drug release	Stainless steel, PCL [76], Hydrogel [77,108,109], 5-layer BD composite [78]	3 h [78], 6 h [106], 9 days [76], >1 mth [108]	Proof of concept [77,78], Animal trials [76,108,109]	Targeted on demand drug delivery	2016, 2018, 2019
Biopsy	Magnetic positioning, less invasive & painful procedure	Plastic shell [80], PDMS, SMA Outriggers [79], Aluminum razor [80,81], Polyurethane [110]	indefinite	Proposal [80], Prototype [79], Ex vivo Animal tissue [110]	Biopsy	2012, 2013, 2014
Optical image	BC, minimal electronics, mobile w/magnetic fields	Ice [78], agarose hydrogel [77]	indefinite	Proof of concept [78], Ex vivo [77]	Less invasive surgery	2016, 2018
Ingestion sensor	Skin-worn receiver patch, signficantly reduced size, gastric fluid powered	Edible adhesives [88], Conventional drug tablet powder [74]	4 min [74]	Commercial product	Medication adherence	2015, 2020
Motility Sensor	Flexiblity & non-battery powered device, dissolvable capsule	Polyamic acid, UV Curable epoxy shell [111], Plastic shell [75]	>10,000 bends [111], 26 h [75]	Proof of concept in vitro & ex vivo [111], in vivo animal trials [75]	Gut motility	2017, 2019
(**B**) IBCs that located in intestines						
Drug Administration	Less painful	Stainless steel, PCL	48 days	Clinical trials [107]	Extended/on extended/on demand targeted drug release	2019
Biopsy	Magnetic position, less invasive & Painful procedure	Plastic shell (Simi et al., 2013), PDMS, SMA outriggers [79], 7075 aluminum razor [80,81], polyurethane [110]	Indefinite	Proposal [80], prototype [79], Ex vivo animal tissue [110]	Biopsy	2012, 2013, 2014
Odometry	Soft & Human compliant, Three-arm design takes stabler & Smoother video	BC rubber wheel & Plastic shell [101]	n/a	Ex vivo [71], Animal testing [101]	Distances between/from landmarks	2015
Spectroscopy (EM Radiation)	Lower manufacturing cost, more accurate & precise results	Non-toxic polycarbonate, shellac coating, BC epoxy	6 to 9 h	Proof of concept, ex vivo trials [109]	Blood detection	2016
Ultrasound Imaging	Eliminates certain mapping challenges	BC epoxy seal [112], Parylene coating [100]	n/a	Ex vivo	Ultrasound endoscopy/mapping	2016, 2018
Endoscopy	Requires a laxative & fasting for 24 h prior to administration	BC plastics, Teflon coating Shell	4–12 h	Commercial product [106]	Endoscopy	2020
(**C**) IBCs that function in whole GI tract						
Electro-chemical Sensing	Comparable to precision lab equipment	Polyimede flexible substrate, Polyether ether ketone shell	72 h [93]	Clinical trials	Chemical markers, Disease diagnosis	2015
Temperature	Reliable measurements no matter patient’s activity	BC polycarbonate, Medical-grade plastic	hrs to mths to indefinite [71,113,114]	Commercial product	Core temperature	2004, 2015, 2020
Pressure	Safety, Reliability, Cost, Size	BC polycarbonate, M3 Crystal (resin)	Hrs [100] to mths to indefinite [75]	Commercial product	Pressure, Gut motility	2018, 2019
pH	Miniature & Powerable by many means	BC polycarbonate, Medical-grade plastic	48 h [114] to 20+ days [71,99]	Commercial product	Gut motility, Stomach acidity, Gastric reflux	2015, 2017, 2020
Gas(CO_2_, O_2_, H_2_)	Linear measurements	BC adhesive, Opaque Polyethelyne Shell	>4 days [68]	Commercial product	Gut disorders (carbohydrate malabsorption, IBS, etc.)	2017
Biomarkers	Modular organic sensor design	Parylene/Epoxy, PDMS Shell	>9 mth [105]	Clinical trials	Bleeding, Infection, Inflammation, etc.	2018

**Table 4:** Summary of indicators in implantable biosensors.

Categories of Biological Information	Detection Target (s)	Application Scenarios	References
Physiological signals	Electrophysiological signal	Heart failure, Epilepsy, Parkinson’s disease	[125,126]
	Temperature	Thermoregulatory disorder	[90,127]
	Motion	Parkinson’s disease	[128]
	Respiratory rate	Asthma	[94,129]
	Optical signal	Blindness, Cataract	[130]
Biochemical signals	DA/AA	Parkinson’s Disease, Schizophrenia, Tics Coprolalia syndrome, Pituitary tumor	[94,131]
	Glucose	Diabetes	[132,133]
	K^+^/Na^+^/Ca^2+^	Stroke	[134]
	pH	Stroke, Traumatic brain injury, Migraine with aura	[64]
Mechanical pressure	Intracranial pressure	Intracranial hypertension, Brain tumor, Brain injury	[135]
	Intraocular pressure	Ocular hypertension, Glaucoma.	[136]
	Pressure in artery	Hypertension	[99,136]
	Intra-abdominal pressure	Abdominal compartment syndrome	[137]
	Intra-bladder pressure	Underactive bladder syndrome.	[138]

**Table 5:** Summary of power sources to three kinds of biosensors.

Sensors	Types of Power		Detected Signals/Targets
Wearable biosensors	Batteries		Glucose [34], Lactate [34,146], pH, Temperature [147], Potassium [146], Li ions [148]
	PV/Solar cells		Pressure [149], Strain [150]
	BFCs		Fructose [151], Glucose [152,153], Exosomes from cell [153], Lactic acid [137,154]
	RF harvesters		Location tracking [155]
	Energy harvesters	PENGs	Pressure [156], Strain [157], Motion [158], Vibration [159]
		TENGs	Tactile [160,161], Breathing, pulse [162], Motion [163,164], Strain [165,166]
		EMGs	Motion [167]
		ESGs	Vibration [168]
		TEGs	Temperature [169,170], Pressure [169], Breathing [171], Humidity, Motion [170]
	Hybrid energy	TENGs& PENGs	Gait, Sweat [172]
		TEGs& PENGs	Temperature, Motion, Pulse [173]
		TENGs& solar cell	Motion [174]
		PV& TEGs	Temperature, Heartbeat, SpO_2_, Body acceleration [175]
Ingestible biosensors	Batteries	AgO or Li or Li-ion batteries	Temperature [71,109], pH [71,136], Gas [100], Pressure [75,100], Distance [71,101], Motility [71,75,78], Image [71,80,104,109], Biomolecules [105]
	BFCs		Temperature [176], Medication adherence [74]
	RF harvesters		Image [177]
	Energy harvesters	PENGs	Motility [78]
		TEGs	Temperature, pH, Iron ions [177]
Implantable biosensors	Batteries	Li batteries	Heart beating [178]
		Li-ion batteries	Neural signals [179]
	PV/Solar cells		Optical signal [130]
	BFCs		Glucose [142], Disaccharide trehalose [180], Temperature [181], Humidity [127]
	Energy harvesters	PENGs	Pressure [182], Vibration [183], Breathing [129], Heart beating [184], Motion [128], Pulse [185], Blood pressure [185]
		TENGs	Fullness of the bladder [138], Breathing [186], Blood pressure [183,187,188]
		EMGs	Heart beating [189,190]

**Table 6:** Information fusion of different information modalities.

Modalities	Sensors/Database	Medical Applications	Fusion Technique	Ref.
Images	The JAFFE, the Cohn-Kanade, and the MMI image	Facial expression identification	Gauss–Laguerre wavelet textural feature fusion	[196]
Electro-Magnetic (EM) tracking and MEMS inertial sensors	A miniature inertial measurement unit and an electromagnetic navigation system	Attitude estimation for laparoscopic surgical tools	An extended Kalman filter	[197]
Inertia and vision	Inertial and visual motion capture sensors	Knee flexion kinematics for functional rehabilitation movements	An extended Kalman filter	[198]
Continuous glucose monitoring (CGM) data	CGM sensors	Blood glucose estimation	A K-mean algorithm	[199]
ECG	MIT-BIH-AR database	Heartbeat classification	A series of one-versus-one SVM binary classifiers	[200]
Acceleration and ventilation	Hip and wrist accelerometers, Respiratory signals from the AB ventilation sensors	Free-living physical activity assessment	SVM algorithm	[201]
Acceleration	Wrist accelerometer	Classification of physical activities	Weighted majority vote, Naïve Bayes combination, and Behavior knowledge space combination	[202]
Daily steps and speed	Wearable or mobile devices	Assessing intensity pattern of lifelogging physical activity	Multiple density map (with Ellipse fitting model to remove irregular uncertainties firstly), Dempster-Shafer theory of evidence (DST)	[203]
MU-POF	Inertial measurement units (IMUs) intensity-variation based Polymer Optical Fiber (POF) curvature sensor	A knee sleeve for monitoring of physical therapy	Multiparameter fusion (MPF) algorithm	[204]
Blood flow waveform (BFW)	Wireless cuffless limbs blood sensors	Cardiovascular patients who have high-risk levels of arteriosclerosis	Multiparameter fusion (MPF) algorithm	[205]
Location	Integration of inertial sensors, Magnetic field and bluetooth low energy (BLE) technologies from the wearable beacon	Identifying low-level micro-activities that can be used to derive complex activities of daily living (ADL) performed by home-care patients	Gathering the location information of the target user by using a wearable beacon embedded with a magnetometer and inertial sensors	[206]
Gait Speed	Wrist-mounted inertial sensors (wrist-mounted accelerometer and barometer)	Accurate, Long, Real-time, Low-power, and Indoor/Outdoor speed estimation in daily life	A personalized model taking unique gait style of each subject into account	[207]
Multi-Frequency Electrical Impedance (MFEI)	Electrical impedance measurements	Improve Radiofrequency Ablation Monitoring (RAM) by monitoring of RFA within multiple tissue types	Non-linear machine learning (ML) models	[208]
IMU	Inertial measurement units (IMUs) securely fixed to body segment	Analyzing hip and knee joint kinematics	Sensor fusion algorithms and a biomechanical model	[209]
IMU	Wearable IMU (inertial measurement units) sensors	Motor fluctuations in patients with Parkinson’s disease (PD)	Time-frequency (TF) representation and multiway data analysis tools (i.e., tensor decomposition)	[210]
Respiration, Cardiac Electrical signals, Blood pressures, SpO_2_	Sensors of respiration, ECG, Blood pressure, Saturation of oxygen	Remote health care systems	Evidence theory	[211]
Antenna signals	Antennas, RFID tags	The automatic online recognition of surgical instruments	Layer model with redundant, complementary, Cooperative signal fusion strategies	[212]
Upper body movements, Force and torque applied to and orientation and position of the surgical instruments	Desktop microphone (audio), Logitech quickcam (bird’s-eye video), Syntek USB Video Capture (surgical field video), ATI mini40 (force and torque), PTI Phoenix VZ3000 (marker locations)	Laparoscopic surgery skill acquisition	The calculation of mechanical energy	[213]
EEG, Electro-oculogram (EOG), EMG	EEG: low wave energy, sleep spindles, K_Complex_, delta, theta, stability; EOG: eye movement, correlation, movement activity; EMG: movement activity	Personalized sleep staging system	Evolutionary algorithm and symbolic fusion	[214]
Joint motion and acceleration	Jaw motion (JM) sensor, Hand gesture (HG) sensor, Accelerometer	Monitoring ingestive behavior	Calculating the product between the JM and HG function, Computing the mean of the acceleration signals	[215]
Time series, Histopathological images, Knowledge databases, Patient histories	Spanning images, Text, Genomics data	Explainable AI for medical diagnosis and health monitor	Graph neural networks	[216]

## Data Availability

Not applicable.

## References

[1] Guo Y., Liu X., Peng S., Jiang X., Xu K., Chen C., Wang Z., Dai C., Chen W. (2020). A review of wearable and unobtrusive sensing technologies for chronic disease management. Comput. Biol. Med..

[2] Libanori R., Erb R.M., Reiser A., Le Ferrand H., Süess M.J., Spolenak R., Studart A.R. (2012). Stretchable heterogeneous composites with extreme mechanical gradients. Nat. Commun..

[3] Liu Y., Pharr M., Salvatore G.A. (2017). Lab-on-Skin: A Review of Flexible and Stretchable Electronics for Wearable Health Monitoring. ACS Nano.

[4] Sun Y., Choi W.M., Jiang H., Huang Y.Y., Rogers J.A. (2006). Controlled buckling of semiconductor nanoribbons for stretchable electronics. Nat. Nanotechnol..

[5] Martirosyan N., Kalani M.Y.S. (2011). Epidermal Electronics. World Neurosurg..

[6] Yamada T., Hayamizu Y., Yamamoto Y., Yomogida Y., Izadi-Najafabadi A., Futaba D.N., Hata K. (2011). A stretchable carbon nanotube strain sensor for human-motion detection. Nat. Nanotechnol..

[7] Wu Q., Qiao Y., Guo R., Naveed S., Hirtz T., Li X., Fu Y., Wei Y., Deng G., Yang Y. (2020). Triode-Mimicking Graphene Pressure Sensor with Positive Resistance Variation for Physiology and Motion Monitoring. ACS Nano.

[8] Gao Q., Li H., Zhang J., Xie Z., Zhang J., Wang L. (2019). Microchannel Structural Design For a Room-Temperature Liquid Metal Based Super-Stretchable Sensor. Sci. Rep..

[9] Yang T., Xie D., Li Z., Zhu H. (2017). Recent Advances in Wearable Tactile Sensors: Materials, Sensing Mechanisms, and Device Performance. Mater. Sci. Eng. R Rep..

[10] Zhong C., Deng Y., Roudsari A.F., Kapetanovic A., Anantram M.P., Rolandi M. (2011). A Polysaccharide Bioprotonic Field-Effect Transistor. Nat. Commun..

[11] Imani S., Bandodkar A.J., Mohan A.M.V., Kumar R., Yu S., Wang J., Mercier P.P. (2016). A Wearable Chemical-Electrophysiological Hybrid Biosensing System for Real-Time Health and Fitness Monitoring. Nat. Commun..

[12] Tang L., Shang J., Jiang X. (2021). Multilayered Electronic Transfer Tattoo That Can Enable the Crease Amplification Effect. Sci. Adv..

[13] Kang B.C., Ha T.J. (2018). Wearable Carbon Nanotube Based Dry-Electrodes for Electrophysiological Sensors. Jpn. J. Appl. Phys..

[14] Sato W., Murata K., Uraoka Y., Shibata K., Yoshikawa S. (2021). Emotional Valence Sensing Using a Wearable Facial EMG Device. Sci. Rep..

[15] Vescio B., Quattrone A., Nisticò R., Crasà M., Quattrone A. (2021). Wearable Devices for Assessment of Tremor. Front. Neurol..

[16] Wu Y.-D., Ruan S.-J., Lee Y.-H. (2021). An Ultra-Low Power Surface EMG Sensor for Wearable Biometric and Medical Applications. Biosensors.

[17] Nielsen J.M., Rades D., Kjaer T.W. (2022). Wearable electroencephalography for ultra-long-term seizure monitoring: A systematic review and future prospects. Expert Rev. Med. Devices.

[18] Al-Qatatsheh A., Morsi Y., Zavabeti A., Zolfagharian A., Salim N., Kouzani A.Z., Mosadegh B., Gharaie S. (2020). Blood Pressure Sensors: Materials, Fabrication Methods, Performance Evaluations and Future Perspectives. Sensors.

[19] Kim J., Chou E.-F., Le J., Wong S., Chu M., Khine M. (2019). Soft Wearable Pressure Sensors for Beat-to-Beat Blood Pressure Monitoring. Adv. Healthc. Mater..

[20] Arakawa T. (2018). Recent Research and Developing Trends of Wearable Sensors for Detecting Blood Pressure. Sensors.

[21] Qiao Y.C., Wang Y.F., Jian J.M., Li M.R., Jiang G.Y., Li X.S., Deng G., Ji S.R., Wei Y.H., Pang Y. (2020). Multifunctional and High-Performance Electronic Skin Based on Silver Nanowires Bridging Graphene. Carbon.

[22] Rachim V.P., Chung W.Y. (2019). Multimodal Wrist Biosensor for Wearable Cuff-Less Blood Pressure Monitoring System. Sci. Rep..

[23] Kim M.K., Kantarcigil C., Kim B., Baruah R.K., Maity S., Park Y., Kim K., Lee S., Malandraki J.B., Avlani S. (2019). Flexible Submental Sensor Patch with Remote Monitoring Controls for Management of Oropharyngeal Swallowing Disorders. Sci. Adv..

[24] Kim K.K., Ha I.H., Kim M., Choi J., Won P., Jo S., Ko S.H. (2020). A Deep-Learned Skin Sensor Decoding the Epicentral Human Motions. Nat. Commun..

[25] Qiao Y., Wang Y., Tian H., Li M., Jian J., Wei Y., Tian Y., Wang D.Y., Pang Y., Geng X. (2018). Multilayer Graphene Epidermal Electronic Skin. ACS Nano.

[26] Tao L.Q., Zhang K.N., Tian H., Liu Y., Wang D.Y., Chen Y.Q., Yang Y., Ren T.L. (2017). Graphene-Paper Pressure Sensor for Detecting Human Motions. ACS Nano.

[27] Tao L.Q., Tian H., Liu Y., Ju Z.Y., Pang Y., Chen Y.Q., Wang D.Y., Tian X.G., Yan J.C., Deng N.Q. (2017). An Intelligent Artificial Throat with Sound-Sensing Ability Based on Laser Induced Graphene. Nat. Commun..

[28] Wei Y., Qiao Y., Jiang G., Wang Y., Wang F., Li M., Zhao Y., Tian Y., Gou G., Tan S. (2019). A Wearable Skinlike Ultra-Sensitive Artificial Graphene Throat. ACS Nano.

[29] Dubey S., Mahnan A., Konczak J. Real-Time Voice Activity Detection Using Neck-Mounted Accelerometers for Controlling a Wearable Vibration Device to Treat Speech Impairment. Proceedings of the 2020 Design of Medical Devices Conference.

[30] Hua G., Miao C., Zhu L., Ming M. Research on the Heart Sound Monitor System of the FBG Intelligent Clothing. Proceedings of the 2011 International Conference on Control, Automation and Systems Engineering.

[31] Shi W.Y., Chiao J.C. (2018). Neural Network Based Real-Time Heart Sound Monitor Using a Wireless Wearable Wrist Sensor. Analog. Integr. Circuits Signal Process..

[32] Mohammadi-Koushki N., Memarzadeh-Tehran H., Goliaei S. A Wearable Device for Continuous Cardiorespiratory System Monitoring. Proceedings of the Conference on Local Computer Networks.

[33] Li S.H., Lin B.S., Tsai C.H., Yang C.T., Lin B.S. (2017). Design of Wearable Breathing Sound Monitoring System for Real-Time Wheeze Detection. Sensors.

[34] Gao W., Emaminejad S., Nyein H.Y.Y., Challa S., Chen K., Peck A., Fahad H.M., Ota H., Shiraki H., Kiriya D. (2016). Fully Integrated Wearable Sensor Arrays for Multiplexed in Situ Perspiration Analysis. Nature.

[35] Abellán-llobregat A., Jeerapan I., Bandodkar A., Vidal L., Canals A., Wang J. (2017). Biosensors and Bioelectronics A Stretchable and Screen-Printed Electrochemical Sensor for Glucose Determination in Human Perspiration. Biosens. Bioelectron..

[36] Anastasova S., Crewther B., Rosa B., Yang G., Bembnowicz P., Curto V., Ip H. (2017). Biosensors and Bioelectronics A Wearable Multisensing Patch for Continuous Sweat Monitoring. Biosens. Bioelectron..

[37] Schazmann B., Morris D., Slater C., Beirne S., Fay C., Reuveny R., Moyna N., Diamond D. (2010). A Wearable Electrochemical Sensor for the Real-Time Measurement of Sweat Sodium Concentration. Anal. Methods.

[38] Yin H., Nyein Y., Gao W., Shahpar Z., Emaminejad S., Challa S., Chen K., Fahad H.M., Tai L., Ota H. (2016). A Wearable Electrochemical Platform for Noninvasive Simultaneous Monitoring of Ca 2+ and PH. ACS Nano.

[39] Windmiller J.R., Bandodkar A.J., Valde G., Parkhomovsky S., Martinez A.G., Wang J. (2012). ChemComm Electrochemical Sensing Based on Printable Temporary Transfer Tattoos. Chem. Commun..

[40] Kinnamon D., Ghanta R., Lin K., Muthukumar S., Prasad S. (2017). Portable Biosensor for Monitoring Cortisol in Low-Volume Perspired Human Sweat. Sci. Rep..

[41] Kim J., Jeerapan I., Imani S., Cho T.N., Bandodkar A., Cinti S., Mercier P.P., Wang J. (2016). Noninvasive Alcohol Monitoring Using a Wearable Tattoo-Based Iontophoretic-Biosensing System. ACS Sens..

[42] Gao W., Nyein H.Y.Y., Shahpar Z., Fahad H.M., Chen K., Emaminejad S., Gao Y., Tai L., Ota H., Wu E. (2016). Wearable Microsensor Array for Multiplexed Heavy Metal Monitoring of Body Fluids. ACS Sens..

[43] Guinovart T., Bandodkar A.J., Windmiller J.R., Andrade F.J., Wang J. (2013). A Potentiometric Tattoo Sensor for Monitoring Ammonium in Sweat. Analyst.

[44] Tierney M.J., Tamada J.A., Potts R.O., Jovanovic L., Garg S. (2001). Clinical Evaluation of the GlucoWatch ® Biographer: A Continual, Non-Invasive Glucose Monitor for Patients with Diabetes. Biosens. Bioelectron..

[45] Bandodkar A.J., Jia W., Yard C., Wang X., Ramirez J., Wang J. (2015). Tattoo-Based Noninvasive Glucose Monitoring: A Proof-of-Concept Study. Anal. Chem..

[46] Li T., Liang B., Ye Z., Zhang L., Xu S., Tu T., Zhang Y., Cai Y., Zhang B., Fang L. (2022). An Integrated and Conductive Hydrogel-Paper Patch for Simultaneous Sensing of Chemical–Electrophysiological Signals. Biosens. Bioelectron..

[47] Yoon S., Yoon H., Zahed M.A., Park C., Kim D., Park J.Y. (2022). Multifunctional Hybrid Skin Patch for Wearable Smart Healthcare Applications. Biosens. Bioelectron..

[48] Sempionatto J.R., Lin M., Yin L., De Lapaz E., Pei K., Sonsa-ard T., de Loyola Silva A.N., Khorshed A.A., Zhang F., Tostado N. (2021). An Epidermal Patch for the Simultaneous Monitoring of Haemodynamic and Metabolic Biomarkers. Nat. Biomed. Eng..

[49] Yang T., Jiang X., Zhong Y., Zhao X., Lin S., Li J., Li X., Xu J., Li Z., Zhu H. (2017). A Wearable and Highly Sensitive Graphene Strain Sensor for Precise Home-Based Pulse Wave Monitoring. ACS Sens..

[50] Randazzo V., Ferretti J., Pasero E. (2020). A Wearable Smart Device to Monitor Multiple Vital Parameters—VITAL ECG. Electronics.

[51] Luo N., Dai W., Li C., Zhou Z., Lu L., Poon C.C.Y., Chen S.C., Zhang Y., Zhao N. (2016). Flexible Piezoresistive Sensor Patch Enabling Ultralow Power Cuffless Blood Pressure Measurement. Adv. Funct. Mater..

[52] Ma X., Ahadian S., Liu S., Zhang J., Liu S., Cao T., Lin W., Wu D., De Barros N.R., Zare M.R. (2021). Smart Contact Lenses for Biosensing Applications. Adv. Intell. Syst..

[53] Heikenfeld J., Jajack A., Rogers J., Gutruf P., Tian L., Pan T., Li R., Khine M., Kim J., Wang J. (2018). Wearable Sensors: Modalities, Challenges, and Prospects. Lab Chip.

[54] Gong S., Schwalb W., Wang Y., Chen Y., Tang Y., Si J., Shirinzadeh B., Cheng W. (2014). A Wearable and Highly Sensitive Pressure Sensor with Ultrathin Gold Nanowires. Nat. Commun..

[55] Lai Y.C., Deng J., Liu R., Hsiao Y.C., Zhang S.L., Peng W., Wu H.M., Wang X., Wang Z.L. (2018). Actively Perceiving and Responsive Soft Robots Enabled by Self-Powered, Highly Extensible, and Highly Sensitive Triboelectric Proximity- and Pressure-Sensing Skins. Adv. Mater..

[56] Luo N., Huang Y., Liu J., Chen S.C., Wong C.P., Zhao N. (2017). Hollow-Structured Graphene–Silicone-Composite-Based Piezoresistive Sensors: Decoupled Property Tuning and Bending Reliability. Adv. Mater..

[57] Choong C.L., Shim M.B., Lee B.S., Jeon S., Ko D.S., Kang T.H., Bae J., Lee S.H., Byun K.E., Im J. (2014). Highly Stretchable Resistive Pressure Sensors Using a Conductive Elastomeric Composite on a Micropyramid Array. Adv. Mater..

[58] Mannsfeld S.C.B., Tee B.C.K., Stoltenberg R.M., Chen C.V.H.H., Barman S., Muir B.V.O., Sokolov A.N., Reese C., Bao Z. (2010). Highly Sensitive Flexible Pressure Sensors with Microstructured Rubber Dielectric Layers. Nat. Mater..

[59] Cheng W., Yu L., Kong D., Yu Z., Wang H., Ma Z., Wang Y., Wang J., Pan L., Shi Y. (2018). Fast-Response and Low-Hysteresis Flexible Pressure Sensor Based on Silicon Nanowires. IEEE Electron. Device Lett..

[60] Pang Y., Zhang K., Yang Z., Jiang S., Ju Z., Li Y., Wang X., Wang D., Jian M., Zhang Y. (2018). Epidermis Microstructure Inspired Graphene Pressure Sensor with Random Distributed Spinosum for High Sensitivity and Large Linearity. ACS Nano.

[61] Wang C., Xia K., Zhang M., Jian M., Zhang Y. (2017). An All-Silk-Derived Dual-Mode E-Skin for Simultaneous Temperature-Pressure Detection. ACS Appl. Mater. Interfaces.

[62] Webb R.C., Bonifas A.P., Behnaz A., Zhang Y., Yu K.J., Cheng H., Shi M., Bian Z., Liu Z., Kim Y.S. (2013). Ultrathin Conformal Devices for Precise and Continuous Thermal Characterization of Human Skin. Nat. Mater..

[63] Zheng X., Zhang C., Chen P., Zhao K., Jiang H., Jiang Z., Pan H., Wang Z., Jia W. (2020). A CRNN System for Sound Event Detection Based on Gastrointestinal Sound Dataset Collected by Wearable Auscultation Devices. IEEE Access.

[64] He X.L., Li D.J., Zhou J., Wang W.B., Xuan W.P., Dong S.R., Jin H., Luo J.K. (2013). High Sensitivity Humidity Sensors Using Flexible Surface Acoustic Wave Devices Made on Nanocrystalline ZnO/Polyimide Substrates. J. Mater. Chem. C.

[65] Kim J., Campbell A.S., de Ávila B.E.-F., Wang J. (2019). Wearable Biosensors for Healthcare Monitoring. Nat. Biotechnol..

[66] Gao W., Nyein H.Y.Y., Shahpar Z., Tai L.C., Wu E., Bariya M., Ota H., Fahad H.M., Chen K., Javey A. Wearable Sweat Biosensors. Proceedings of the Technical Digest—International Electron Devices Meeting.

[67] Koh A., Kang D., Xue Y., Lee S., Pielak R.M., Kim J., Hwang T., Min S., Banks A., Bastien P. (2016). A Soft, Wearable Microfluidic Device for the Capture, Storage, and Colorimetric Sensing of Sweat. Sci. Transl. Med..

[68] Munje R.D., Muthukumar S., Jagannath B., Prasad S. (2017). A New Paradigm in Sweat Based Wearable Diagnostics Biosensors Using Room Temperature Ionic Liquids (RTILs). Sci. Rep..

[69] Wang L., Jackman J.A., Ng W.B., Cho N.J. (2016). Flexible, Graphene-Coated Biocomposite for Highly Sensitive, Real-Time Molecular Detection. Adv. Funct. Mater..

[70] Fu Y., Wang N., Yang A., Law H.K.-W., Li L., Yan F. (2017). Highly Sensitive Detection of Protein Biomarkers with Organic Electrochemical Transistors. Adv. Mater..

[71] Min J., Yang Y., Wu Z., Gao W. (2020). Robotics in the Gut. Adv. Ther..

[72] Bettinger C.J. (2015). Materials Advances for Next-Generation Ingestible Electronic Medical Devices. Trends Biotechnol..

[73] Yoshida S., Miyaguchi H., Nakamura T. Proof of Concept for Tablet-Shaped Ingestible Core-Body Thermometer with Gastric Acid Battery. Proceedings of the 2019 IEEE 1st Global Conference on Life Sciences and Technologies.

[74] Hafezi H., Robertson T.L., Moon G.D., Au-Yeung K.Y., Zdeblick M.J., Savage G.M. (2015). An Ingestible Sensor for Measuring Medication Adherence. IEEE Trans. Biomed. Eng..

[75] Benken A., Gianchandani Y. (2019). PassiveWireless Pressure Sensing for Gastric Manometry. Micromachines.

[76] Abramson A., Caffarel-Salvador E., Khang M., Dellal D., Silverstein D., Gao Y., Frederiksen M., Vegge A., Hubálek F., Water J. (2019). An Ingestible Self-Orienting System for Oral Delivery of Macromolecules. Science.

[77] D’Argentre A.D.P., Perry S., Iwata Y., Iwasaki H., Iwase E., Fabozzo A., Will I., Rus D., Damian D.D., Miyashita S. Programmable Medicine: Autonomous, Ingestible, Deployable Hydrogel Patch and Plug for Stomach Ulcer Therapy. Proceedings of the 2018 IEEE International Conference on Robotics and Automation (ICRA).

[78] Miyashita S., Guitron S., Yoshida K., Li S., Damian D.D., Rus D. Ingestible, Controllable, and Degradable Origami Robot for Patching Stomach Wounds. Proceedings of the 2016 IEEE International Conference on Robotics and Automation (ICRA).

[79] Kong K., Yim S., Choi S., Jeon D. (2012). A Robotic Biopsy Device for Capsule Endoscopy. J. Med. Device..

[80] Simi M., Gerboni G., Menciassi A., Valdastri P. (2013). Magnetic Torsion Spring Mechanism for a Wireless Biopsy Capsule. J. Med. Device..

[81] Lin Z.H., Hsu W.S., Preet A., Yeh L.H., Chen Y.H., Pao Y.P., Lin S.F., Lee S., Fan J.C., Wang L. (2021). Ingestible Polysaccharide Battery Coupled with a Self-Charging Nanogenerator for Controllable Disinfection System. Nano Energy.

[82] Gao C., Bai C., Gao J., Xiao Y., Han Y., Shaista A., Zhao Y., Qu L. (2020). A Directly Swallowable and Ingestible Microsupercapacitor. J. Mater. Chem. A Mater..

[83] Moglia A., Menciassi A., Schurr M.O., Dario P. (2007). Wireless Capsule Endoscopy: From Diagnostic Devices to Multipurpose Robotic Systems. Biomed. Microdevices.

[84] Zhang Y., Yang H., Yang D., Liu X., Liu Z. (2020). Polynomial Profile Optimization Method of a Magnetic Petal-Shaped Capsule Robot. Mechatronics.

[85] Sendoh M., Ishiyama K., Arai K.I. Fabrication of Magnetic Actuator for Use in Capsule Endoscope. Proceedings of the Program of the 2003 IEEE International Magnetics Conference.

[86] Chen X., Yu J., Wu Z., Meng Y., Kong S. (2020). Toward a Maneuverable Miniature Robotic Fish Equipped with a Novel Magnetic Actuator System. IEEE Trans Syst. Man. Cybern. Syst..

[87] Liu X., Yang Y., Inda M.E., Lin S., Wu J., Kim Y., Chen X., Ma D., Lu T.K., Zhao X. (2021). Magnetic Living Hydrogels for Intestinal Localization, Retention, and Diagnosis. Adv. Funct. Mater..

[88] Liu J., Sugiyama H., Nakayama T., Miyashita S. Magnetic Sensor Based Topographic Localization for Automatic Dislocation of Ingested Button Battery. Proceedings of the 2020 IEEE International Conference on Robotics and Automation (ICRA).

[89] Zhang Y.H., Shepard K.L. A 0.6-Mm2 Powering and Data Telemrtry System Compatible Ultrasound B-Mode Imaging for Freely Moving Biomedical Sensor System. Proceedings of the 2019 IEEE Custom Integrated Circuits Conference (CICC).

[90] Monge M., Lee-Gosselin A., Shapiro M.G., Emami A. (2017). Localization of Microscale Devices in Vivo Using Addressable Transmitters Operated as Magnetic Spins. Nat. Biomedical. Eng..

[91] Wu X., Hou W., Peng C., Zheng X., Fang X., He J. (2008). Wearable Magnetic Locating and Tracking System for MEMS Medical Capsule. Sens. Actuators A Phys..

[92] Lee M.M., Lee E.-M., Cho B.L., Eshraghian K., Kim Y.-H. (2006). The UTCOMS: A Wireless Video Capsule Nanoendoscope. Endosc. Microsc..

[93] Mc Caffrey C., Twomey K., Ogurtsov V.I. (2015). Development of a Wireless Swallowable Capsule with Potentiostatic Electrochemical Sensor for Gastrointestinal Track Investigation. Sens. Actuators B Chem..

[94] Wang A., Banerjee S., Barth B.A., Bhat Y.M., Chauhan S., Gottlieb K.T., Konda V., Maple J.T., Murad F., Pfau P.R. (2000). Wireless Capsule Endoscopy. Gastrointest. Endosc..

[95] Freitas R.B., Rodrigues J.A., Puga H., Correia J.H. (2018). Design, Simulation, and Fabrication of an Ingestible Capsule with Gastric Balloon for Obesity Treatment. Biomed. Phys. Eng. Express.

[96] Liu S., Chu S., Beardslee L.A., Ghodssi R. (2020). Hybrid and Passive Tissue-Anchoring Mechanism for Ingestible Resident Devices. J. Microelectromechanical Syst..

[97] Spada C., Shah S.K., Riccioni M.E., Spera G., Marchese M., Iacopini F., Familiari P., Costamagna G. (2007). Video Capsule Endoscopy in Patients with Known or Suspected Small Bowel Stricture Previously Tested with the Dissolving Patency Capsule. J. Clin. Gastroenterol..

[98] Godovskii Y.K., Levin V.Y., Slonimskii G.L., Zhdanov A.A., Andrianov K.A. (1969). Micro-Calorimetric Study of Polydimethylsiloxane (PDMS). Polym. Sci. U.S.S.R..

[99] Kalantar-Zadeh K., Ha N., Ou J.Z., Berean K.J. (2017). Ingestible Sensors. ACS Sens..

[100] Kalantar-Zadeh K., Berean K.J., Ha N., Chrimes A.F., Xu K., Grando D., Ou J.Z., Pillai N., Campbell J.L., Brkljača R. (2018). A Human Pilot Trial of Ingestible Electronic Capsules Capable of Sensing Different Gases in the Gut. Nat. Electron..

[101] Karargyris A., Koulaouzidis A. (2015). OdoCapsule: Next-Generation Wireless Capsule Endoscopy with Accurate Lesion Localization and Video Stabilization Capabilities. IEEE Trans Biomed. Eng..

[102] Kuo B., Maneerattanaporn M., Lee A.A., Baker J.R., Wiener S.M., Chey W.D., Wilding G.E., Hasler W.L. (2011). Generalized Transit Delay on Wireless Motility Capsule Testing in Patients with Clinical Suspicion of Gastroparesis, Small Intestinal Dysmotility, or Slow Transit Constipation. Dig. Dis. Sci..

[103] Li F., Gurudu S.R., De Petris G., Sharma V.K., Shiff A.D., Heigh R.I., Fleischer D.E., Post J., Erickson P., Leighton J.A. (2008). Retention of the Capsule Endoscope: A Single-Center Experience of 1000 Capsule Endoscopy Procedures. Gastrointest. Endosc..

[104] Eliakim R. (2017). Where Do I See Minimally Invasive Endoscopy in 2020: Clock Is Ticking. Ann. Transl. Med..

[105] Mimee M., Nadeau P., Hayward A., Carim S., Flanagan S., Jerger L., Collins J., McDonnell S., Swartwout R., Citorik R.J. (2018). An Ingestible Bacterial-Electronic System to Monitor Gastrointestinal Health. Science.

[106] Liu X., Steiger C., Lin S., Parada G.A., Liu J., Chan H.F., Yuk H., Phan N.V., Collins J., Tamang S. (2019). Ingestible Hydrogel Device. Nat. Commun..

[107] EI-Said I., Aboelwafa A., Khali R., ElGazayerly O. (2016). Baclofen Novel Gastroretentive Extended Release Gellan Gum Superporous Hydrogel Hybrid System: In Vitro and in Vivo Evaluation. Drug Deliv..

[108] Scudellari M. Shot to the Gut: “Robotic” Pill Sails through Human Safety Study. IEEE Spectrum. https://spectrum.ieee.org/shot-to-the-gut-robotic-pill-sails-through-human-safety-study.

[109] Schostek S., Zimmermann M., Keller J., Fode M., Melbert M., Schurr M.O., Gottwald T., Prosst R.L. (2016). Telemetric Real-Time Sensor for the Detection of Acute Upper Gastrointestinal Bleeding. Biosens. Bioelectron..

[110] Yim S., Gultepe E., Gracias D.H., Sitti M. (2014). Biopsy Using a Magnetic Capsule Endoscope Carrying, Releasing, and Retrieving Untethered Microgrippers. IEEE Trans Biomed. Eng..

[111] Dagdeviren C., Javid F., Joe P., Von Erlach T., Bensel T., Wei Z., Saxton S., Cleveland C., Booth L., McDonnell S. (2017). Flexible Piezoelectric Devices for Gastrointestinal Motility Sensing. Nat. Biomed. Eng..

[112] Lay H.S., Qiu Y., Al-Rawhani M., Beeley J., Poltarjonoks R., Seetohul V., Cumming D., Cochran S., Cummins G., Desmulliez M.P.Y. Progress towards a Multi-Modal Capsule Endoscopy Device Featuring Microultrasound Imaging. Proceedings of the IEEE International Ultrasonics Symposium.

[113] McKenzie J.E., Osgood D.W. (2004). Validation of a New Telemetric Core Temperature Monitor. J. Therm. Biol..

[114] Koziolek M., Grimm M., Becker D., Iordanov V., Zou H., Shimizu J., Wanke C., Garbacz G., Weitschies W. (2015). Investigation of PH and Temperature Profiles in the GI Tract of Fasted Human Subjects Using the Intellicap(®) System. J. Pharm. Sci..

[115] Demosthenous P., Pitris C., Georgiou J. (2016). Infrared Fluorescence-Based Cancer Screening Capsule for the Small Intestine. IEEE Trans. Biomed. Circuits Syst..

[116] Kong Y., Zou X., McCandler C., Kirtane A., Ning S., Zhou J., Abid A., Jafari M., Rogner J., Langer R. (2019). 3D-Printed Gastric Resident Electronics. Adv. Mater. Technol..

[117] Bellinger A., Jafari M., Grant T., Zhang S., Slater H., Wenger E., Mo S., Lee Y., Mazdiyasni H., Eckhoff P. (2016). Oral, Ultra-Long-Lasting Drug Delivery: Application toward Malaria Elimination Goals. Sci. Transl. Med..

[118] Lee S., Reuveny A., Reeder J., Lee S., Jin H., Liu Q., Yokota T., Sekitani T., Isoyama T., Abe Y. (2016). A Transparent Bending-Insensitive Pressure Sensor. Nat. Nanotechnol..

[119] Frost M., Meyerhoff M.E. (2006). In Vivo Chemical Sensors: Tackling Biocompatibility. Anal. Chem..

[120] Thomé T., Erhardt M.C.G., Leme A.A., Al Bakri I., Bedran-Russo A.K., Bertassoni L.E. (2015). Emerging Polymers in Dentistry.

[121] Wo Y., Brisbois E.J., Bartlett R.H., Meyerhoff M.E. (2016). Recent Advances in Thromboresistant and Antimicrobial Polymers for Biomedical Applications: Just Say Yes to Nitric Oxide (NO). Biomater. Sci..

[122] Anderson J.M., Jones J.A. (2007). Phenotypic Dichotomies in the Foreign Body Reaction. Biomaterials.

[123] Polikov V.S., Tresco P.A., Reichert W.M. (2005). Response of Brain Tissue to Chronically Implanted Neural Electrodes. J. Neurosci. Methods.

[124] Soto R.J., Hall J.R., Brown M.D., Taylor J.B., Schoenfisch M.H. (2017). In Vivo Chemical Sensors: Role of Biocompatibility on Performance and Utility. Anal. Chem..

[125] Goyal A., Goetz S., Stanslaski S., Oh Y., Rusheen A.E., Klassen B., Miller K., Blaha C.D., Bennet K.E., Lee K. (2021). The Development of an Implantable Deep Brain Stimulation Device with Simultaneous Chronic Electrophysiological Recording and Stimulation in Humans. Biosens. Bioelectron..

[126] Turan M., Almalioglu Y., Araujo H., Konukoglu E., Sitti M. (2018). Deep EndoVO: A Recurrent Convolutional Neural Network (RCNN) Based Visual Odometry Approach for Endoscopic Capsule Robots. Neurocomputing.

[127] Shoji K., Akiyama Y., Suzuki M., Nakamura N., Ohno H., Morishima K. (2016). Biofuel Cell Backpacked Insect and Its Application to Wireless Sensing. Biosens. Bioelectron..

[128] Dagdeviren C., Yang B.D., Su Y., Tran P.L., Joe P., Anderson E., Xia J., Doraiswamy V., Dehdashti B., Feng X. (2014). Conformal Piezoelectric Energy Harvesting and Storage from Motions of the Heart, Lung, and Diaphragm. Proc. Natl. Acad. Sci. USA.

[129] Li Z., Zhu G., Yang R., Wang A.C., Wang Z.L. (2010). Muscle-Driven in Vivo Nanogenerator. Adv. Mater..

[130] Wu C.Y., Kuo P.H., Lin P.K., Chen P.C., Sung W.J., Ohta J., Tokuda T., Noda T. (2018). A CMOS 256-Pixel Photovoltaics-Powered Implantable Chip with Active Pixel Sensors and Iridium-Oxide Electrodes for Subretinal Prostheses. Sens. Mater..

[131] Zhang B., Li C., Zhang H., Chen Y., Jiang H., Chen L., Ur Rehman F., Wang X. (2018). In Vivo Dopamine Biosensor Based on Copper(I) Sulfide Functionalized Reduced Graphene Oxide Decorated Microelectrodes. J. Biomed. Nanotechnol..

[132] Hao J., Xiao T., Wu F., Yu P., Mao L. (2016). High Antifouling Property of Ion-Selective Membrane: Toward in Vivo Monitoring of PH Change in Live Brain of Rats with Membrane-Coated Carbon Fiber Electrodes. Anal. Chem..

[133] Heller J., Helwing R., Baker R., Tutte M. (1983). Controlled Release of Water-Soluble Macromolecules from Bioerodible Hydrogel. Biomaterials.

[134] Odijk M., Van Der Wouden E.J., Olthuis W., Ferrari M.D., Tolner E.A., Van Den Maagdenberg A.M.J.M., Van Den Berg A. (2015). Microfabricated Solid-State Ion-Selective Electrode Probe for Measuring Potassium in the Living Rodent Brain: Compatibility with DC-EEG Recordings to Study Spreading Depression. Sens. Actuators B Chem..

[135] Chen L.Y., Tee B.C.K., Chortos A.L., Schwartz G., Tse V., Lipomi D.J., Wong H.S.P., McConnell M.V., Bao Z. (2014). Continuous Wireless Pressure Monitoring and Mapping with Ultra-Small Passive Sensors for Health Monitoring and Critical Care. Nat. Commun..

[136] Boutry C.M., Beker L., Kaizawa Y., Vassos C., Tran H., Hinckley A.C., Pfattner R., Niu S., Li J., Claverie J. (2019). Biodegradable and Flexible Arterial-Pulse Sensor for the Wireless Monitoring of Blood Flow. Nat. Biomed. Eng..

[137] Xu X., He Z., Shan Y., Mao Q., Feng J., Gao G. (2021). Intra-Abdominal Pressure Measurements in Neurocritical Patients. J. Vis. Exp..

[138] Arab Hassani F., Mogan R.P., Gammad G.G.L., Wang H., Yen S.C., Thakor N.V., Lee C. (2018). Toward Self-Control Systems for Neurogenic Underactive Bladder: A Triboelectric Nanogenerator Sensor Integrated with a Bistable Micro-Actuator. ACS Nano.

[139] Horton J.G., Adams D.L. (2005). The Cortical Column: A Structure without a Function. Philos. Trans. R. Soc. B Biol. Sci..

[140] Guan S., Wang J., Gu X., Zhao Y., Hou R., Fan H., Zou L., Gao L., Du M., Li C. (2019). Elastocapillary Self-Assembled Neurotassels for Stable Neural Activity Recordings. Sci. Adv..

[141] Pu Z., Tu J., Han R., Zhang X., Wu J., Fang C., Wu H., Zhang X., Yu H., Li D. (2018). A Flexible Enzyme-Electrode Sensor with Cylindrical Working Electrode Modified with a 3D Nanostructure for Implantable Continuous Glucose Monitoring. Lab Chip.

[142] Cinquin P., Gondran C., Giroud F., Mazabrard S., Pellissier A., Boucher F., Alcaraz J.P., Gorgy K., Lenouvel F., Mathé S. (2010). A Glucose BioFuel Cell Implanted in Rats. PLoS ONE.

[143] Van Loon J., Mars P. (1997). Biocompatibility: The Latest Developments. Med. Device Technol..

[144] (2009). Biological Evaluation of Medical Devices—Part 1: Evaluation and Testing within a Risk Management Process.

[145] Mani S., Vediyappan V., Chen S.M., Madhu R., Pitchaimani V., Chang J.Y., Liu S. (2016). Bin Hydrothermal Synthesis of NiWO4 Crystals for High Performance Non-Enzymatic Glucose Biosensors. Sci. Rep..

[146] Sempionatto J.R., Nakagawa T., Pavinatto A., Mensah S.T., Imani S., Mercier P., Wang J. (2017). Eyeglasses Based Wireless Electrolyte and Metabolite Sensor Platform. Lab Chip.

[147] Yokus M.A., Songkakul T., Pozdin V.A., Bozkurt A., Daniele M.A. (2020). Wearable Multiplexed Biosensor System toward Continuous Monitoring of Metabolites. Biosens. Bioelectron..

[148] Hanitra I.N., Lobello L., Stradolini F., Tuoheti A., Criscuolo F., Kilic T., Demarchi D., Carrara S., De Micheli G. A Flexible Front-End for Wearable Electrochemical Sensing. Proceedings of the 2018 IEEE International Symposium on Medical Measurements and Applications.

[149] Choi J., Kwon D., Kim B., Kang K., Gu J., Jo J., Na K., Ahn J., Del Orbe D., Kim K. (2020). Wearable Self-Powered Pressure Sensor by Integration of Piezo-Transmittance Microporous Elastomer with Organic Solar Cell. Nano Energy.

[150] Li C., Cong S., Tian Z., Song Y., Yu L., Lu C., Shao Y., Li J., Zou G., Rümmeli M.H. (2019). Flexible Perovskite Solar Cell-Driven Photo-Rechargeable Lithium-Ion Capacitor for Self-Powered Wearable Strain Sensors. Nano Energy.

[151] Bollella P., Boeva Z., Latonen R.M., Kano K., Gorton L., Bobacka J. (2021). Highly Sensitive and Stable Fructose Self-Powered Biosensor Based on a Self-Charging Biosupercapacitor. Biosens. Bioelectron..

[152] Huang J., Zhang Y., Ding F., Chen D., Wang Y., Jin X., Zhu X. (2021). Rational Design of Electroactive Redox Enzyme Nanocapsules for High-Performance Biosensors and Enzymatic Biofuel Cell. Biosens. Bioelectron..

[153] Gu C., Bai L., Pu L., Gai P., Li F. (2021). Highly Sensitive and Stable Self-Powered Biosensing for Exosomes Based on Dual Metal-Organic Frameworks Nanocarriers. Biosens. Bioelectron..

[154] Sempionatto J.R., Raymundo-Pereira P.A., Azeredo N.N.F., De Loyola Silva A.E.A., Angnes L., Wang J. (2020). Enzymatic Biofuel Cells Based on Protective Hydrophobic Carbon Paste Electrodes: Towards Epidermal Bioenergy Harvesting in the Acidic Sweat Environment. Chem. Commun..

[155] Vital D., Bhardwaj S., Volakis J.L. (2020). Textile-Based Large Area RF-Power Harvesting System for Wearable Applications. IEEE Trans Antennas Propag..

[156] Kar E., Bose N., Dutta B., Mukherjee N., Mukherjee S. (2019). Ultraviolet-and Microwave-Protecting, Self-Cleaning e-Skin for Efficient Energy Harvesting and Tactile Mechanosensing. ACS Appl. Mater. Interfaces.

[157] Song H.B., Karakurt I., Wei M., Liu N., Chu Y., Zhong J., Lin L. (2018). Lead Iodide Nanosheets for Piezoelectric Energy Conversion and Strain Sensing. Nano Energy.

[158] Qiu Y., Zhang H., Hu L., Yang D., Wang L., Wang B., Ji J., Liu G., Liu X., Lin J. (2012). Flexible Piezoelectric Nanogenerators Based on ZnO Nanorods Grown on Common Paper Substrates. Nanoscale.

[159] Bairagi S., Ghosh S., Ali S.W. (2020). A Fully Sustainable, Self-Poled, Bio-Waste Based Piezoelectric Nanogenerator: Electricity Generation from Pomelo Fruit Membrane. Sci. Rep..

[160] Chen J., Chen B., Han K., Tang W., Wang Z.L. (2019). A Triboelectric Nanogenerator as a Self-Powered Sensor for a Soft–Rigid Hybrid Actuator. Adv. Mater. Technol..

[161] Lai Y.C., Hsiao Y.C., Wu H.M., Wang Z.L. (2019). Waterproof Fabric-Based Multifunctional Triboelectric Nanogenerator for Universally Harvesting Energy from Raindrops, Wind, and Human Motions and as Self-Powered Sensors. Adv. Sci..

[162] Zhao G., Zhang Y., Shi N., Liu Z., Zhang X., Wu M., Pan C., Liu H., Li L., Wang Z.L. (2019). Transparent and Stretchable Triboelectric Nanogenerator for Self-Powered Tactile Sensing. Nano Energy.

[163] Wen Z., Yang Y., Sun N., Li G., Liu Y., Chen C., Shi J., Xie L., Jiang H., Bao D. (2018). A Wrinkled PEDOT:PSS Film Based Stretchable and Transparent Triboelectric Nanogenerator for Wearable Energy Harvesters and Active Motion Sensors. Adv. Funct. Mater..

[164] Zhang R., Hummelgård M., Örtegren J., Yang Y., Andersson H., Balliu E., Blomquist N., Engholm M., Olsen M., Wang Z.L. (2019). Sensing Body Motions Based on Charges Generated on the Body. Nano Energy.

[165] Lan L., Yin T., Jiang C., Li X., Yao Y., Wang Z., Qu S., Ye Z., Ping J., Ying Y. (2019). Highly Conductive 1D-2D Composite Film for Skin-Mountable Strain Sensor and Stretchable Triboelectric Nanogenerator. Nano Energy.

[166] He T., Shi Q., Wang H., Wen F., Chen T., Ouyang J., Lee C. (2019). Beyond Energy Harvesting—Multi-Functional Triboelectric Nanosensors on a Textile. Nano Energy.

[167] Wang N., Han R., Chen C., Gu J., Li X. Double-Deck Metal Solenoids 3D Integrated in Silicon Wafer for Kinetic Energy Harvesters. Proceedings of the 2021 5th IEEE Electron Devices Technology and Manufacturing Conference.

[168] Lu Y., Marty F., Galayko D., Laheurte J.M., Basset P. (2018). A Power Supply Module for Autonomous Portable Electronics: Ultralow-Frequency MEMS Electrostatic Kinetic Energy Harvester with a Comb Structure Reducing Air Damping. Microsyst. Nanoeng..

[169] Zhang F., Zang Y., Huang D., Di C.A., Zhu D. (2015). Flexible and Self-Powered Temperature-Pressure Dual-Parameter Sensors Using Microstructure-Frame-Supported Organic Thermoelectric Materials. Nat. Commun..

[170] Yuan J., Zhu R. Self-Powered Wearable Multi-Sensing Bracelet with Flexible Thermoelectric Power Generator. Proceedings of the 2019 20th International Conference on Solid-State Sensors, Actuators and Microsystems & Eurosensors XXXIII (TRANSDUCERS & EUROSENSORS XXXIII).

[171] Xue H., Yang Q., Wang D., Luo W., Wang W., Lin M., Liang D., Luo Q. (2017). A Wearable Pyroelectric Nanogenerator and Self-Powered Breathing Sensor. Nano Energy.

[172] Zhu M., Shi Q., He T., Yi Z., Ma Y., Yang B., Chen T., Lee C. (2019). Self-Powered and Self-Functional Cotton Sock Using Piezoelectric and Triboelectric Hybrid Mechanism for Healthcare and Sports Monitoring. ACS Nano.

[173] Zhu P., Wang Y., Sheng M., Wang Y., Yu Y., Deng Y. (2019). A Flexible Active Dual-Parameter Sensor for Sensitive Temperature and Physiological Signal Monitoring: Via Integrating Thermoelectric and Piezoelectric Conversion. J. Mater. Chem. A Mater..

[174] Wen Z., Yeh M.H., Guo H., Wang J., Zi Y., Xu W., Deng J., Zhu L., Wang X., Hu C. (2016). Self-Powered Textile for Wearable Electronics by Hybridizing Fiber-Shaped Nanogenerators, Solar Cells, and Supercapacitors. Sci. Adv..

[175] Mohsen S., Zekry A., Youssef K., Abouelatta M. (2021). A Self-Powered Wearable Wireless Sensor System Powered by a Hybrid Energy Harvester for Healthcare Applications. Wirel. Pers. Commun..

[176] Nadeau P., El-Damak D., Glettig D., Kong Y.L., Mo S., Cleveland C., Booth L., Roxhed N., Langer R., Chandrakasan A.P. (2017). Prolonged Energy Harvesting for Ingestible Devices. Nat. Biomed. Eng..

[177] Koulaouzidis A., Iakovidis D.K., Karargyris A., Rondonotti E. (2015). Wireless Endoscopy in 2020: Will It Still Be a Capsule?. World J. Gastroenterol..

[178] Scrosati B. (2011). History of Lithium Batteries. J. Solid State Electrochem..

[179] Li P., Principe J.C., Bashirullah R. A Wireless Power Interface for Rechargeable Battery Operated Neural Recording Implants. Proceedings of the Annual International Conference of the IEEE Engineering in Medicine and Biology Society.

[180] Rasmussen M., Ritzmann R.E., Lee I., Pollack A.J., Scherson D. (2012). An Implantable Biofuel Cell for a Live Insect. J. Am. Chem. Soc..

[181] Bollella P., Lee I., Blaauw D., Katz E. (2020). A Microelectronic Sensor Device Powered by a Small Implantable Biofuel Cell. ChemPhysChem.

[182] Nguyen T.D., Curry E.J. Biodegradable Piezoelectric Sensor. Proceedings of the 2019 IEEE 16th International Conference on Wearable and Implantable Body Sensor Networks.

[183] Wang Z.L., Song J. (2006). Piezoelectric Nanogenerators Based on Zinc Oxide Nanowire Arrays. Science.

[184] Jeong C.K., Han J.H., Palneedi H., Park H., Hwang G.T., Joung B., Kim S.G., Shin H.J., Kang I.S., Ryu J. (2017). Comprehensive Biocompatibility of Nontoxic and High-Output Flexible Energy Harvester Using Lead-Free Piezoceramic Thin Film. APL Mater..

[185] Cheng X., Xue X., Ma Y., Han M., Zhang W., Xu Z., Zhang H., Zhang H. (2016). Implantable and Self-Powered Blood Pressure Monitoring Based on a Piezoelectric Thinfilm: Simulated, in Vitro and in Vivo Studies. Nano Energy.

[186] Zheng Q., Shi B., Fan F., Wang X., Yan L., Yuan W., Wang S., Liu H., Li Z., Wang Z.L. (2014). In Vivo Powering of Pacemaker by Breathing-Driven Implanted Triboelectric Nanogenerator. Adv. Mater..

[187] Liu Z., Ma Y., Ouyang H., Shi B., Li N., Jiang D., Xie F., Qu D., Zou Y., Huang Y. (2019). Transcatheter Self-Powered Ultrasensitive Endocardial Pressure Sensor. Adv. Funct. Mater..

[188] Fan F.R., Tian Z.Q., Lin Wang Z. (2012). Flexible Triboelectric Generator. Nano Energy.

[189] Zurbuchen A., Haeberlin A., Bereuter L., Pfenniger A., Bosshard S., Kernen M., Philipp Heinisch P., Fuhrer J., Vogel R. (2018). Endocardial Energy Harvesting by Electromagnetic Induction. IEEE Trans. Biomed. Eng..

[190] Haeberlin A., Rosch Y., Tholl M.V., Gugler Y., Okle J., Heinisch P.P., Reichlin T., Burger J., Zurbuchen A. (2020). Intracardiac Turbines Suitable for Catheter-Based Implantation—An Approach to Power Battery and Leadless Cardiac Pacemakers?. IEEE Trans. Biomed. Eng..

[191] Hosaka T., Kubota K., Hameed A.S., Komaba S. (2020). Research Development on K-Ion Batteries. Chem. Rev..

[192] Kim Y.J., Wu W., Sang-Eun C., Whitacre J.F., Bettinger C.J. (2017). Biologically Derived Melanin Electrodes in Aqueous Sodium-Ion Energy Storage Devices. Proc. Natl. Acad. Sci. USA.

[193] Veeralingam S., Khandelwal S., Badhulika S. (2020). AI/ML-Enabled 2-D-RuS 2 Nanomaterial-Based Multifunctional, Low Cost, Wearable Sensor Platform for Non-Invasive Point of Care Diagnostics. IEEE Sens. J..

[194] Zhang M., Feng H., Luo H., Li Z., Zhang X. (2020). Comfort and Health Evaluation of Live Mutton Sheep during the Transportation Based on Wearable Multi-Sensor System. Comput. Electron. Agric..

[195] Haque A., Milstein A., Fei-Fei L. (2020). Illuminating the Dark Spaces of Healthcare with Ambient Intelligence. Nature.

[196] Poursaberi A., Noubari H.A., Gavrilova M., Yanushkevich S.N. (2012). Gauss-Laguerre Wavelet Textural Feature Fusion with Geometrical Information for Facial Expression Identification. EURASIP J. Image Video Process..

[197] Ren H., Kazanzides P. Hybrid Attitude Estimation for Laparoscopic Surgical Tools: A Preliminary Study. Proceedings of the 31st Annual International Conference of the IEEE Engineering in Medicine and Biology Society: Engineering the Future of Biomedicine, EMBC.

[198] Tannous H., Istrate D., Benlarbi-Delai A., Sarrazin J., Gamet D., Ho Ba Tho M.C., Dao T.T. (2016). A New Multi-Sensor Fusion Scheme to Improve the Accuracy of Knee Flexion Kinematics for Functional Rehabilitation Movements. Sensors.

[199] Xiong F., Hipszer B.R., Joseph J., Kam M. (2011). Improved Blood Glucose Estimation through Multi-Sensor Fusion. Proceedings of the Annual International Conference of the IEEE Engineering in Medicine and Biology Society. EMBS.

[200] Zhang Z., Luo X. (2014). Heartbeat Classification Using Decision Level Fusion. Biomed. Eng. Lett..

[201] Liu S., Gao R.X., John D., Staudenmayer J.W., Freedson P.S. (2012). Multisensor Data Fusion for Physical Activity Assessment. IEEE Trans. Biomed. Eng..

[202] Chowdhury A.K., Tjondronegoro D., Chandran V., Trost S.G. (2017). Ensemble Methods for Classification of Physical Activities from Wrist Accelerometry. Med. Sci. Sports Exerc..

[203] Qi J., Yang P., Hanneghan M., Tang S. (2017). Multiple Density Maps Information Fusion for Effectively Assessing Intensity Pattern of Lifelogging Physical Activity. Neurocomputing.

[204] Vargas-Valencia L.S., Schneider F.B.A., Leal-Junior A.G., Caicedo-Rodriguez P., Sierra-Arevalo W.A., Rodriguez-Cheu L.E., Bastos-Filho T., Frizera-Neto A. (2020). Sleeve for Knee Angle Monitoring: An IMU-POF Sensor Fusion System. IEEE J. Biomed. Health Inform..

[205] Yuan Z., Huang X., Wan P., Zhao C., Zhang Y., Zhang B., Wang J., Zhang H., Sang S. (2021). A Cost-Effective Smartphone-Based Device for Ankle-Brachial Index (ABI) Detection. Comput. Methods Programs Biomed..

[206] Sridharan M., Bigham J., Campbell P.M., Phillips C., Bodanese E. (2020). Inferring Micro-Activities Using Wearable Sensing for ADL Recognition of Home-Care Patients. IEEE J. Biomed. Health Inform..

[207] Soltani A., Dejnabadi H., Savary M., Aminian K. (2020). Real-World Gait Speed Estimation Using Wrist Sensor: A Personalized Approach. IEEE J. Biomed. Health Inform..

[208] Besler E., Wang Y.C., Sahakian A.V. (2020). Early and Late Fusion Machine Learning on Multi-Frequency Electrical Impedance Data to Improve Radiofrequency Ablation Monitoring. IEEE J. Biomed. Health Inform..

[209] Bastiaansen B.J.C., Wilmes E., Brink M.S., de Ruiter C.J., Savelsbergh G.J.P., Steijlen A., Jansen K.M.B., van der Helm F.C.T., Goedhart E.A., van der Laan D. (2020). An Inertial Measurement Unit Based Method to Estimate Hip and Knee Joint Kinematics in Team Sport Athletes on the Field. J. Vis. Exp..

[210] Ghoraani B., Hssayeni M.D., Bruack M.M., Jimenez-Shahed J. (2020). Multilevel Features for Sensor-Based Assessment of Motor Fluctuation in Parkinson’s Disease Subjects. IEEE J. Biomed. Health Inform..

[211] Sung W.T., Chang K.Y. (2013). Evidence-Based Multi-Sensor Information Fusion for Remote Health Care Systems. Sens. Actuators A Phys..

[212] Neumuth T., Meißner C. (2012). Online Recognition of Surgical Instruments by Information Fusion. Int. J. Comput. Assist. Radiol. Surg..

[213] Anderson F., Birch D.W., Boulanger P., Bischof W.F. (2012). Sensor Fusion for Laparoscopic Surgery Skill Acquisition. Comput. Aided Surg..

[214] Chen C., Ugon A., Zhang X., Amara A., Garda P., Ganascia J.G., Philippe C., Pinna A. Personalized Sleep Staging System Using Evolutionary Algorithm and Symbolic Fusion. Proceedings of the Annual International Conference of the IEEE Engineering in Medicine and Biology Society.

[215] Fontana J.M., Farooq M., Sazonov E. (2014). Automatic Ingestion Monitor: A Novel Wearable Device for Monitoring of Ingestive Behavior. IEEE Trans. Biomed. Eng..

[216] Holzinger A., Malle B., Saranti A., Pfeifer B. (2021). Towards Multi-Modal Causability with Graph Neural Networks Enabling Information Fusion for Explainable AI. Inf. Fusion.

[217] Banerjee H., Ponraj G., Kirthika S.K., Suman M.V., Lim C.M., Ren H. (2019). Hydrogel-Shielded Soft Tactile Sensor for Biocompatible Drug Delivery Monitoring. J. Med. Device..

[218] Belknap R., Weis S., Brookens A., Au-Yeung K.Y., Moon G., DiCarlo L., Reves R. (2013). Feasibility of an Ingestible Sensor-Based System for Monitoring Adherence to Tuberculosis Therapy. PLoS ONE.

